# CD8^+^ T cells maintain killing of MHC-I-negative tumor cells through the NKG2D–NKG2DL axis

**DOI:** 10.1038/s43018-023-00600-4

**Published:** 2023-08-03

**Authors:** Emily C. Lerner, Karolina I. Woroniecka, Vincent M. D’Anniballe, Daniel S. Wilkinson, Aditya A. Mohan, Selena J. Lorrey, Jessica Waibl-Polania, Lucas P. Wachsmuth, Alexandra M. Miggelbrink, Joshua D. Jackson, Xiuyu Cui, Jude A. Raj, William H. Tomaszewski, Sarah L. Cook, John H. Sampson, Anoop P. Patel, Mustafa Khasraw, Michael D. Gunn, Peter E. Fecci

**Affiliations:** 1 Duke University School of Medicine, Durham, NC USA; 2 Department of Biomedical Engineering, Duke University, Durham, NC USA; 3 Department of Pathology, Duke University Medical Center, Durham, NC USA; 4 Preston Robert Tisch Brain Tumor Center, Duke University Medical Center, Durham, NC USA; 5 Department of Neurosurgery, Duke University Medical Center, Durham, NC USA; 6 Department of Immunology, Duke University Medical Center, Durham, NC USA; 7 Center for Advanced Genomic Technologies, Duke University, Durham, NC USA; 8 Department of Medicine, Duke University Medical Center, Durham, NC USA

**Keywords:** Tumour immunology, Cancer immunotherapy, Cancer therapeutic resistance, CNS cancer, Cancer

## Abstract

The accepted paradigm for both cellular and anti-tumor immunity relies upon tumor cell killing by CD8^+^ T cells recognizing cognate antigens presented in the context of target cell major histocompatibility complex (MHC) class I (MHC-I) molecules. Likewise, a classically described mechanism of tumor immune escape is tumor MHC-I downregulation. Here, we report that CD8^+^ T cells maintain the capacity to kill tumor cells that are entirely devoid of MHC-I expression. This capacity proves to be dependent instead on interactions between T cell natural killer group 2D (NKG2D) and tumor NKG2D ligands (NKG2DLs), the latter of which are highly expressed on MHC-loss variants. Necessarily, tumor cell killing in these instances is antigen independent, although prior T cell antigen-specific activation is required and can be furnished by myeloid cells or even neighboring MHC-replete tumor cells. In this manner, adaptive priming can beget innate killing. These mechanisms are active in vivo in mice as well as in vitro in human tumor systems and are obviated by NKG2D knockout or blockade. These studies challenge the long-advanced notion that downregulation of MHC-I is a viable means of tumor immune escape and instead identify the NKG2D–NKG2DL axis as a therapeutic target for enhancing T cell-dependent anti-tumor immunity against MHC-loss variants.

## Abstract (web summary)

Fecci and colleagues show that tumor cells having lost MHC-I, a major mechanism of immune escape, are amenable to killing by CD8^+^ T cells through an MHC-I-independent, alternative pathway via NKG2D and NKG2DL interaction and granzyme.

## Main

The long-accepted paradigm for adaptive anti-tumor cellular immunity relies on antigen-specific tumor targeting by activated CD8^+^ T cells. CD8^+^ T cell cytotoxicity, in turn, is classically believed to depend upon T cell receptor (TCR) recognition of tumor antigens presented exclusively in the context of cell surface major histocompatibility complex (MHC) class I (MHC-I) molecules. A critical component of the antigen-presentation function of MHC-I is β-2 microglobulin (β2m), which is conserved across all classical MHC-I alleles present in mice and humans. In turn, mutations in β2m leading to decreased or absent MHC-I expression are purported to constitute a common mechanism by which tumors are able to evade T cell responses, rendering them immunologically ‘cold’ (refs. ^1–4^). The frequency of such MHC-I downregulation varies by tumor histology, with certain tumors, such as glioblastoma, often expressing little to no MHC-I^5,6^.

Recent preclinical and clinical studies, however, have demonstrated somewhat mixed roles for MHC-I and β2m in dictating responses to cancer immune-based platforms, such as immune checkpoint blockade (ICB). While some have suggested that resistance to ICB emerges through inactivation of tumor antigen presentation^7–9^, for instance, others have implied that low tumor β2m and MHC-I expression is instead associated with favorable prognosis^10,11^. These mixed findings highlight the need for further investigation into the role of tumor MHC-I as well as revisiting traditional notions of anti-tumor immunity.

Following our recent investigations into T cell exhaustion and ICB resistance in tumors^12,13^, we sought to better evaluate the role of MHC-I expression within the tumor microenvironment (TME). Herein, we reveal that T cell-activating immunotherapies remain effective against glioma and melanoma lines engineered to lack MHC-I expression. Such efficacy proves independent of natural killer (NK) cells and, instead, relies consistently upon CD8^+^ T cell-mediated cytotoxicity, despite the absence of MHC-I on tumor targets. This cytotoxicity is both antigen and MHC agnostic and, instead, is mediated by T cell NKG2D engagement of non-classical MHC-I NKG2D ligands (NKG2DLs) on tumor cells. Subsequent tumor cell killing depends on prior TCR activation (albeit even by an irrelevant antigen), revealing that adaptive priming can beget subsequent innate killing. Tumor cell killing appears to involve cytotoxic degranulation but is not dependent on Fas. This cytotoxicity mechanism is active in vivo in mice as well as in vitro in human cells and is required for killing of MHC-negative tumor cells even in tumors with heterogeneous MHC expression. Such findings challenge the traditional model of T cell-mediated tumor cell killing and likewise provide a therapeutic blueprint for licensing immune responses in MHC-loss tumor variants.

## Results

### Immunotherapy for MHC-I-negative tumors requires CD8^+^ T cells

**Fig. 1:**
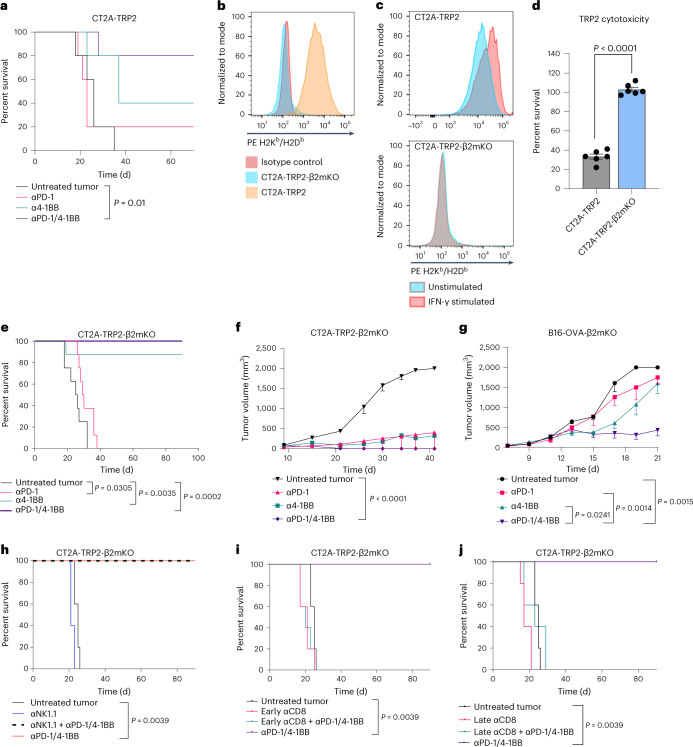
Immunotherapies remain effective against tumor cells lacking MHC-I in a CD8^+^ T cell-dependent manner. **a**, Kaplan–Meier survival curve of mice implanted with CT2A-TRP2 tumors i.c. and then treated with anti-PD-1, anti-4-1BB or αPD-1/4-1BB therapy or PBS vehicle control (untreated tumor) (*n* = 5 per group). α, anti. **b**, Flow cytometry of tumor cells stained for H2K^b^ and H2D^b^, the HLA haplotypes expressed in C57BL/6 mice. **c**, Flow cytometry of tumors stained for H2K^b^ and H2D^b^ following 24 h of in vitro stimulation with IFN-γ (20 ng ml^−1^). **d**, Percent survival of CT2A-TRP2 tumor cells or CT2A-TRP2-β2mKO tumor cells co-cultured with tumor-specific TRP2-specific CD8^+^ T cells (TRP2 T cells) (10:1 effector:target ratio, *n* = 6 co-cultures per group). After 24 h of co-culture, the number of remaining viable tumor cells was quantified using flow cytometry, and percent survival was calculated in comparison to values from tumor-only wells. An effector:target ratio curve is shown in Extended Data Fig. 1a. **e**, Kaplan–Meier survival curve of C57BL/6 mice implanted with CT2A-TRP2-β2mKO tumors i.c. and then treated with anti-PD-1, anti-4-1BB or αPD-1/4-1BB therapy or PBS vehicle control (*n* = 8 per group). **f**,**g**, Subcutaneous tumor growth curves of CT2A-TRP2-β2mKO (**f**) and B16-OVA-β2mKO (**g**) tumors implanted into C57BL/6 mice (*n* = 5 per group). **h**, Kaplan–Meier survival curve of mice implanted with CT2A-TRP2-β2mKO tumors i.c. in the presence or absence of NK depletion with anti-NK1.1 therapy (*n* = 5 per group). **i**,**j**, Kaplan–Meier survival curves of mice implanted with CT2A-TRP2-β2mKO tumors i.c. followed by CD8^+^ T depletion with anti-CD8α therapy in the early priming stage (**i**) (day 3 after tumor implantation) or the later effector stage (**j**) (day 7 after tumor implantation). Data in **d**, **f** and **g** are shown as mean ± s.e.m. The *P* value in **d** was calculated with a two-tailed Student’s *t*-test. Tumor growth curves in **f** and **g** were compared with two-way ANOVA and Tukey’s multiple-comparison test. Survival curves in **a**, **e** and **h**–**j** were assessed by the two-sided Gehan–Breslow–Wilcoxon test. The experiments in **a**, **d**, **e** and **g**–**i** were repeated independently at least two times with similar results. Source data

We have previously demonstrated the efficacy of the combination of 4-1BB agonism and anti-programmed cell death protein 1 (PD-1) checkpoint blockade (αPD-1/4-1BB) in a murine CT2A glioma model. Unsurprisingly, such efficacy proved dependent on the presence of CD8^+^ T cells^12^. More recently, to examine the antigen-specific response, we engineered a CT2A mouse glioma tumor line to express tyrosinase-related peptide 2 (TRP2), a weakly immunogenic model antigen, creating the CT2A-TRP2 line. The combination αPD-1/4-1BB therapy demonstrated similar efficacy against orthotopically implanted CT2A-TRP2 tumors, resulting in 80% long-term survival (Fig. 1a).

With initial intent to examine the impact of tumor antigen presentation on facets of the TME, we used CRISPR to knock out (KO) the gene encoding β2m in the CT2A-TRP2 line, generating the CT2A-TRP2-β2mKO line (Fig. 1b). CT2A-TRP2-β2mKO cells lacked MHC-I expression (H2K^b^ and H2D^b^) as assessed by flow cytometry. MHC-I KO was further confirmed with an interferon (IFN)-γ stimulation assay^13^. IFN-γ stimulation resulted in upregulation of H2K^b^ and H2D^b^ in parental CT2A-TRP2 cells but not in CT2A-TRP2-β2mKO cells (Fig. 1c). Additionally, TRP2 TCR-transduced CD8^+^ T cells (TRP2 T cells) failed to kill CT2A-TRP2-β2mKO tumors in vitro but efficiently killed parental CT2A-TRP2 tumors, providing functional validation of MHC-I knockdown in the CT2A-TRP2-β2mKO line (Fig. 1d).

Surprisingly, we found that mice harboring intracranial (i.c.) CT2A-TRP2-β2mKO tumors remained responsive to immunotherapy, demonstrating 100% long-term survival in response to combination αPD-1/4-1BB treatment and 90% long-term survival in response to anti-4-1BB monotherapy (Fig. 1e). As with CT2A-TRP2 tumors, the CT2A-TRP2-β2mKO tumor was uniformly fatal without treatment. Immunotherapeutic efficacy against CT2A-TRP2-β2mKO tumors was not specific to the i.c. compartment, as αPD-1/4-1BB remained effective against this MHC-I-negative glioma when implanted subcutaneously as well (Fig. 1f). Furthermore, αPD-1/4-1BB retained efficacy against orthotopically implanted melanomas lacking MHC-I (B16-F10-OVA-β2mKO, referred to hereafter as B16-OVA-β2mKO) (Fig. 1g). Ultimately, immunotherapeutic success in MHC-I-negative tumors was restricted neither to gliomas nor to the i.c. compartment.

**Extended Data Fig. 1:**
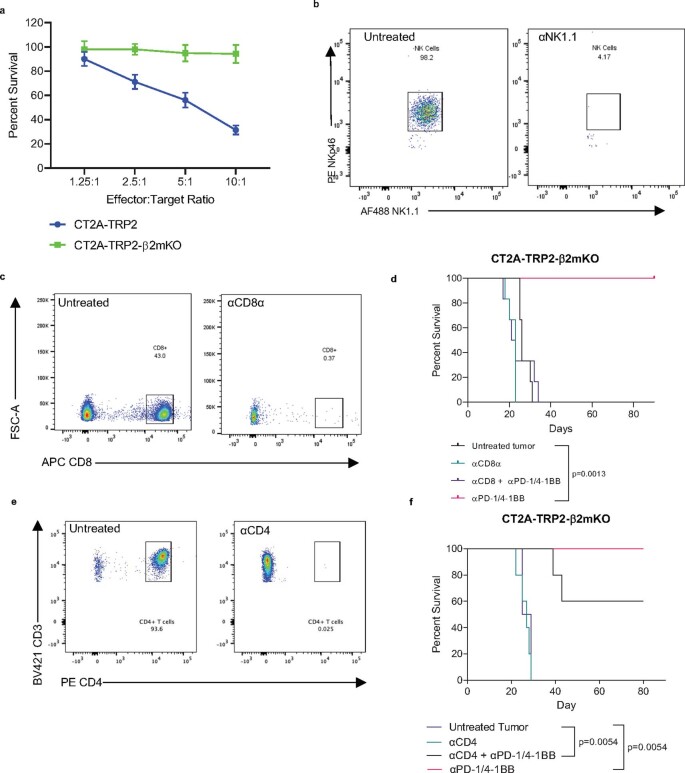
CD8^+^ T cells are required for the success of immunotherapy against *in vivo* MHC-I-negative tumors. **a**. ET ratio curve of percent survival of CT2A-TRP2 tumors or CT2A-TRP2-β2mKO tumors co-cultured with tumor specific TRP-2-specific CD8^+^ T cells (TRP2 T cells) for 24 hours (n = 7 co-cultures/group for each condition). Data in a are shown as mean ± SEM. **b**. In vivo depletion validation of NK cells from blood of tumor bearing mouse. Depletion >99% by count. **c**. In vivo depletion validation of CD8^+^ T cells from blood of tumor bearing mouse. Depletion >99% by count. **d**. Kaplan-Meier survival curve of mice implanted with CT2A-TRP2-β2mKO IC then treated with αPD-1/4-1BB in the presence or absence of CD8 depletion with αCD8α (n = 6 mice/group). **e**. In vivo depletion validation of CD4^+^ T cells from blood of tumor bearing mouse. Depletion >99% by count. **f**. Mice were implanted with CT2A-TRP2-β2mKO IC then treated with αPD-1/4-1BB in the presence or absence of CD4^+^ T cell depletion with αCD4 (untreated n = 4, CD4 depletion n = 5, αPD-1/4-1BB n = 5, αPD-1/4-1BB + CD4 depletion n = 5). Antibody depletion for **d** and **f** was started three days prior to tumor implantation, with maintenance depletion continued for the duration of the experiment. Survival in **a**, **d** & **f** was assessed by two-sided Gehan-Breslow-Wilcoxon test. Experiments in **a**, **d**, & **f** were repeated independently two times with similar results. ET, Effector:target. Source data

Intrigued, we sought to determine the immune cell population responsible for the efficacy of immunotherapy against MHC-I-negative tumors. As NK cells are known to eliminate cells with absent or reduced MHC^14^, we first investigated by depleting NK cells before implanting CT2A-TRP2-β2mKO tumors (Extended Data Fig. 1b). Even in the absence of NK cells, αPD-1/4-1BB still elicited 100% long-term survival (Fig. 1h). We therefore explored whether immunotherapeutic efficacy against CT2A-TRP2-β2mKO tumors might still be dependent on CD8^+^ T cells, even in the absence of tumor MHC-I expression. Depletion of CD8^+^ T cells (Extended Data Fig. 1c) completely abrogated the survival benefit of αPD-1/4-1BB in mice bearing CT2A-TRP2-β2mKO tumors. This proved true whether CD8^+^ T cells were depleted before tumor implantation (Extended Data Fig. 1d) or at early (day 3) or late (day 7) time points after tumor implantation (Fig. 1i,j). Of note, CD4^+^ T cell depletion had a modest but not significant impact on survival (Extended Data Fig. 1e,f).

### Antigen-specific killing persists in MHC-I-negative tumors

**Fig. 2:**
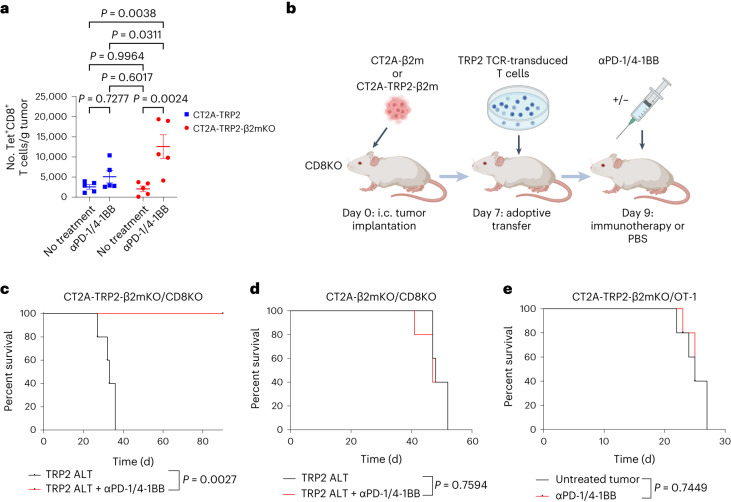
Antigen-specific killing persists in the absence of tumor MHC-I. **a**, Absolute counts of tumor-specific TRP2 CD8^+^ T cells per gram of tumor from C57BL/6 mice bearing either i.c. CT2A-TRP2 or CT2A-TRP2-β2mKO tumors. Mice were implanted with CT2A-TRP2-β2mKO or CT2A-TRP2 tumors i.c. and then treated with i.p. αPD-1/4-1BB at day 18. Tumors were then collected 24 h later, dissociated, stained for the TRP2 tetramer and assessed by flow cytometry (*n* = 5 mice per group). Data are from one of two independent experiments with similar results. Data are shown as mean ± s.e.m. **b**, Schematic of the experimental design. **c**,**d**, Kaplan–Meier survival curves of CD8^+^ T cell global KO mice (CD8KO) implanted i.c. with CT2A-TRP2-β2mKO (*n* = 5 mice per group) (**c**) or CT2A-β2mKO (lacking TRP2 expression) (*n* = 5 mice per group) (**d**) tumors. Mice were treated with 10 × 10^6^ TRP2 TCR-engineered T cells on day 7, followed by i.p. αPD-1/4-1BB therapy or vehicle control. **e**, Kaplan–Meier survival curve of OT-1 mice implanted with CT2A-TRP2-β2mKO tumors, which do not express the OVA antigen, creating a model with few to no tumor-specific T cells present (*n* = 5 mice per group). The *P* value for **a** was calculated with one-way ANOVA and post hoc Tukey’s test. Survival in **c**–**e** was assessed by the two-sided Gehan–Breslow–Wilcoxon test. Source data

**Extended Data Fig. 2:**
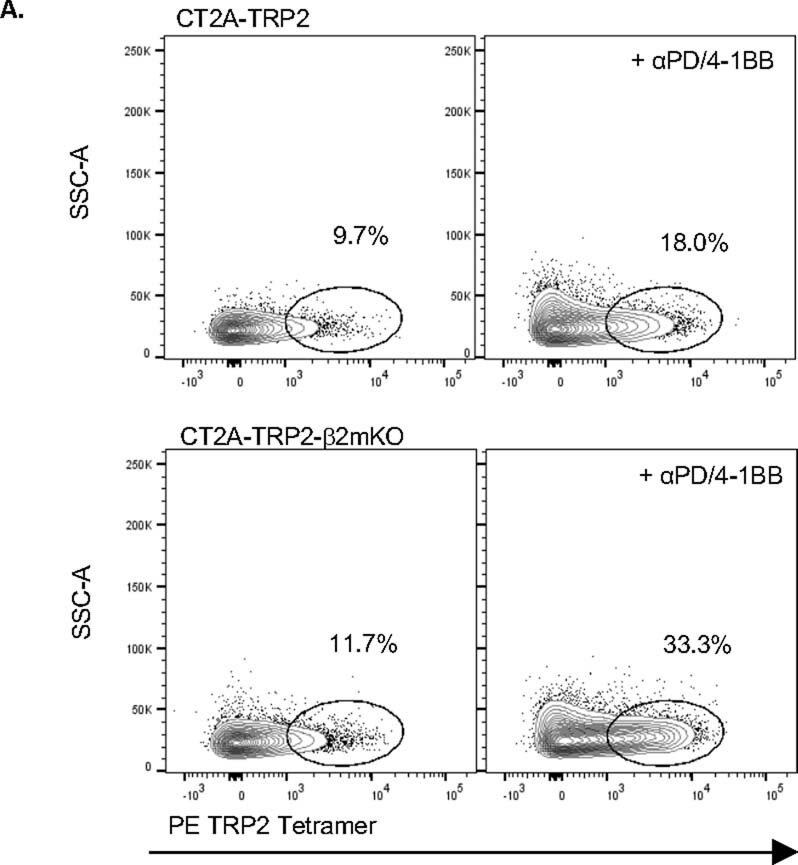
TRP2 Tetramer staining. **a**. TRP2 tetramer staining of CD8^+^ T cells isolated from IC CT2A-TRP2 or CT2A-TRP2-β2mKO tumors. Full tumor infiltrating lymphocyte gating strategy is shown in Extended Data Fig. 4k.

Continued dependence on CD8^+^ T cells raised the question as to whether cytotoxic killing of MHC-I-negative tumors still occurs in an antigen-specific fashion. Interestingly, we noted that αPD-1/4-1BB-treated mice bearing MHC-I-negative CT2A-TRP2-β2mKO tumors accumulated a greater number of TRP2-specific CD8^+^ T cells in the tumor than similarly treated mice bearing MHC-I-positive CT2A-TRP2 tumors (Fig. 2a and Extended Data Fig. 2a). To test the role of antigen specificity, we implanted CT2A-TRP2-β2mKO or CT2A-β2mKO tumors (lacking both TRP2 and MHC-I expression) i.c. into mice lacking CD8^+^ T cells (CD8KO). Mice subsequently received an adoptive transfer of TRP2-specific CD8^+^ T cells (TRP2 ALT), producing a T cell compartment exclusively specific for TRP2 (Fig. 2b). TRP2 ALT in combination with αPD-1/4-1BB was sufficient to eliminate 100% of CT2A-TRP2-β2mKO tumors (Fig. 2c) but was uniformly unsuccessful against CT2A-β2mKO tumors lacking TRP2 expression (Fig. 2d). Additionally, we implanted CT2A-TRP2-β2mKO tumors i.c. into transgenic OT-1 mice. The majority of T cells in these mice recognize residues 257–264 of the ovalbumin (OVA) peptide (OVA_257–264_, SIINFEKL) in the context of H2K^b^ but possess no reactivity against TRP2. Combination αPD-1/4-1BB was completely ineffective against CT2A-TRP2-β2mKO tumors in these mice (Fig. 2e), further suggesting that tumor-specific T cells remain necessary, even in the absence of tumor MHC-I antigen presentation.

**Fig. 3:**
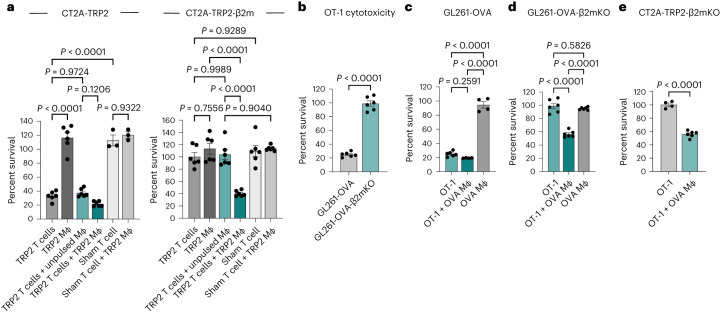
In the absence of tumor MHC-I, CD8^+^ T cell killing requires antigen-specific activation by APCs in vitro. **a**, Left, TRP2 T cells were co-cultured with TRP2 macrophages (Mϕ), unpulsed macrophages and CellTrace Violet-stained tumor cells at a 10:1 T cell:CT2A-TRP2 tumor cell ratio and a 5:1 macrophage:tumor cell ratio. After 24 h of co-culture, the number of remaining viable tumor cells was quantified using flow cytometry, and percent survival was calculated in comparison to tumor cell-only wells (*n* = 6 co-cultures per group). Sham-transduced T cells (sham T cells) were used as a control (*n* = 3 co-cultures per group). Right, groups are as stated but with CT2A-TRP2-β2M tumor cells (*n* = 6 co-cultures per group). **b**, OVA SIINFEKL peptide (257–264)-specific OT-1 T cells were co-cultured with a murine glioma model expressing OVA antigen (GL261-OVA) or MHC-I-negative GL261-OVA-β2mKO tumor cells for 24 h at a 10:1 effector:target ratio. Percent survival was calculated as described above (*n* = 6 co-cultures per group). OVA Mϕ, OVA-pulsed macrophage. **c**, GL261-OVA tumor cells were co-cultured with OT-1 T cells alone (*n* = 6 co-cultures) or OVA-pulsed macrophages alone (*n* = 4 co-cultures) or both (*n* = 4 co-cultures), and percent survival after 24 h was calculated (10:1 T cell:tumor cell ratio and 5:1 macrophage:tumor cell ratio). **d**, As in **c**, but with GL261-OVA-β2mKO tumor cells (all *n* = 6 co-cultures). **e**, CT2A-TRP2-β2mKO tumor cells were co-cultured with non-tumor-specific OT-1 CD8^+^ T cells (OT-1 T cells) alone (*n* = 4 co-cultures) or OT-1 T cells and OVA-pulsed macrophages (*n* = 6 co-cultures) at a 10:1 T cell:tumor cell ratio and a 5:1 macrophage:tumor cell ratio. All data are shown as mean ± s.e.m. *P* values were determined by two-tailed, unpaired Student’s *t*-test for **b** and **e** and by one-way ANOVA with post hoc Tukey’s test for **a**, **c** and **d**. Experiments in **a**–**e** were repeated independently at least two times with similar results. Source data

Above, we demonstrated that TRP2 T cells failed to kill CT2A-TRP2-β2mKO tumor cells in vitro (Fig. 1d), confirming that CD8^+^ T cells alone cannot kill MHC-I-negative tumors, even in the presence of a cognate antigen. We hypothesized then that an additional cell population must be facilitating the observed cytotoxicity in vivo. In vivo, infiltrating myeloid cells constitute the bulk of immune cells within the brain TME^15^. We therefore examined whether adding bone marrow-derived macrophages (BMDMs) would license in vitro killing of CT2A-TRP2-β2mKO tumors by TRP2 T cells. CT2A-TRP2 or CT2A-TRP2-β2mKO (Fig. 3a) tumor cells were cultured with TRP2 T cells in the presence of either TRP2 peptide-loaded BMDMs (TRP2 macrophages) or unpulsed BMDMs (unpulsed macrophages). While TRP2 T cells alone (but not TRP2 macrophages) efficiently killed MHC-I-positive CT2A-TRP2 tumors, neither TRP2 T cells alone nor TRP2 macrophages alone proved capable of killing MHC-I-negative CT2A-TRP2-β2mKO tumors. Remarkably, however, the combination of TRP2 macrophages and TRP2 T cells was indeed sufficient to kill CT2A-TRP2-β2mKO tumors in vitro. Interestingly, the combination of unpulsed macrophages and TRP2 T cells also failed to result in significant tumor cell killing, suggesting that antigen-cognate interactions between macrophages and CD8^+^ T cells are necessary for deriving cytotoxicity against tumor cells lacking MHC-I. Sham TCR-transduced CD8^+^ T cells failed to kill either tumor cell line, even in the presence of TRP2-loaded macrophages. Importantly, we recapitulated these findings in an additional murine glioma line (GL261) expressing a different target antigen (OVA) using OVA-specific OT-1 T cells and OVA-pulsed macrophages (Fig. 3b–d).

While these results suggested the importance of antigen-cognate interactions between antigen-presenting cells (APCs) and T cells, the role of such interactions between T cells and tumor cells lacking MHC-I remained less clear. Therefore, we performed in vitro cytotoxicity experiments using antigen-matched OT-1 CD8^+^ T cells and OVA-pulsed macrophages co-cultured with OVA-negative CT2A-TRP2-β2mKO tumor cells. We found that the combination of OT-1 T cells and OVA-pulsed macrophages was sufficient to kill CT2A-TRP2-β2mKO tumor cells, even though the tumor itself expressed neither MHC-I nor the cognate OVA antigen (Fig. 3e). These data suggest that, following antigen-specific TCR activation, CD8^+^ T cells kill MHC-I-negative tumors via both an antigen-independent and tumor MHC-I-independent mechanism.

### CD8^+^ T cells kill MHC-I-negative tumors via direct contact

**Fig. 4:**
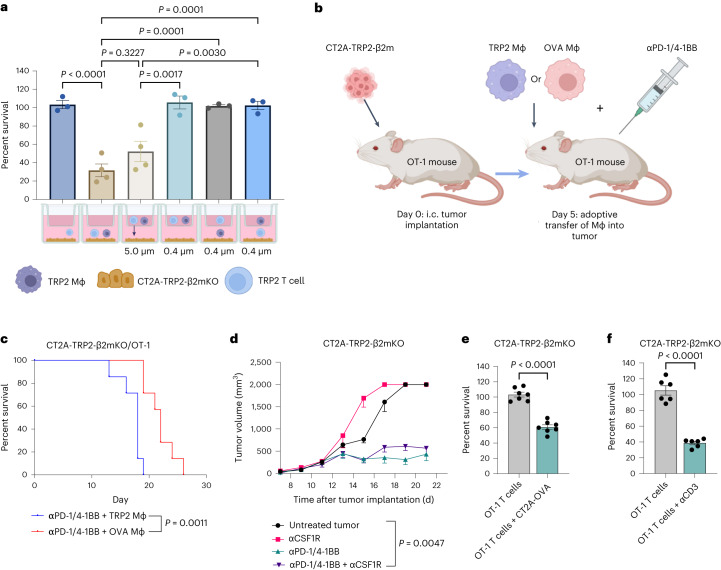
CD8^+^ T cells require contact with APCs and subsequently tumor cells to mediate cytotoxicity against MHC-I-negative tumors. **a**, CT2A-TRP2-β2mKO tumor cells were co-cultured with TRP2 CD8^+^ T cells and TRP2 macrophages in different combinations on either side of a 5.0-μm or a 0.4-μm Transwell as depicted. After 24 h of co-culture, the number of remaining viable tumor cells was quantified using flow cytometry, and percent survival was calculated in comparison to tumor cell-only wells. Cells were plated at a 10:1 T cell:tumor cell ratio and a 5:1 macrophage:tumor cell ratio (*n* = 4 co-cultures per group for all three cell types together and 5.0-μm Transwell groups; all others, *n* = 3 co-cultures per group). **b**, Schematic of the experimental design. **c**, CT2A-TRP2-β2mKO tumors were i.c. implanted into OT-1 mice. Cognate antigen-loaded OVA-pulsed macrophages (5 × 10^5^) or non-cognate antigen-loaded TRP2 macrophages (5 × 10^5^) were then i.c. implanted into the tumor site at day 5 followed by αPD-1/4-1BB immunotherapy. Mice were evaluated for overall survival in the days following tumor implantation (*n* = 5 per group). **d**, Subcutaneous tumor growth curve of B16-OVA-β2mKO tumors implanted into C57BL/6 mice treated with αPD-1/4-1BB in the presence or absence of macrophage depletion with anti-CSF1R therapy (*n* = 5 per group). **e**, Percent survival of CT2A-TRP2-β2mKO tumor cells after 24 h of co-culture with OT-1 T cells either in the presence or the absence of OVA antigen stimulation provided by MHC-I-positive CT2A-OVA tumors at a 10:1 effector:target ratio. Percent survival was calculated as described above (*n* = 7 co-cultures per group). **f**, Percent survival of CT2A-TRP2-β2mKO tumor cells co-cultured with OT-1 T cells in the presence or absence of stimulating anti-CD3 antibody (10:1 effector:target ratio, *n* = 6 co-cultures per group). Data in **a** and **d**–**f** are shown as mean ± s.e.m. *P* values were determined using one-way ANOVA with post hoc Tukey’s test for **a** and by two-tailed, unpaired Student’s *t*-test for **e** and **f**. Tumor growth curves in **d** were analyzed with two-way ANOVA and Tukey’s multiple-comparison test. Survival in **c** was assessed by the two-sided Gehan–Breslow–Wilcoxon test. Experiments in **a** and **c**–**f** were repeated independently at least two times with similar results. Source data

We next examined the mechanistic requirements for killing tumor cells that lack MHC-I. To determine first whether a soluble factor might be involved, we used 0.4-μm Transwell plates, which allow cytokines and other soluble mediators, but not cells, to pass through freely. MHC-I-negative CT2A-TRP2-β2mKO tumor cells were placed on the bottom of the Transwell plates, with various combinations of TRP2 T cells and TRP2 macrophages added to either the same or opposite side of the membrane. Physically separating T cells and/or macrophages from tumor cells obviated tumoricidal activity, suggesting a requirement for direct cell–cell contact rather than a soluble factor (Fig. 4a).

**Extended Data Fig. 3:**
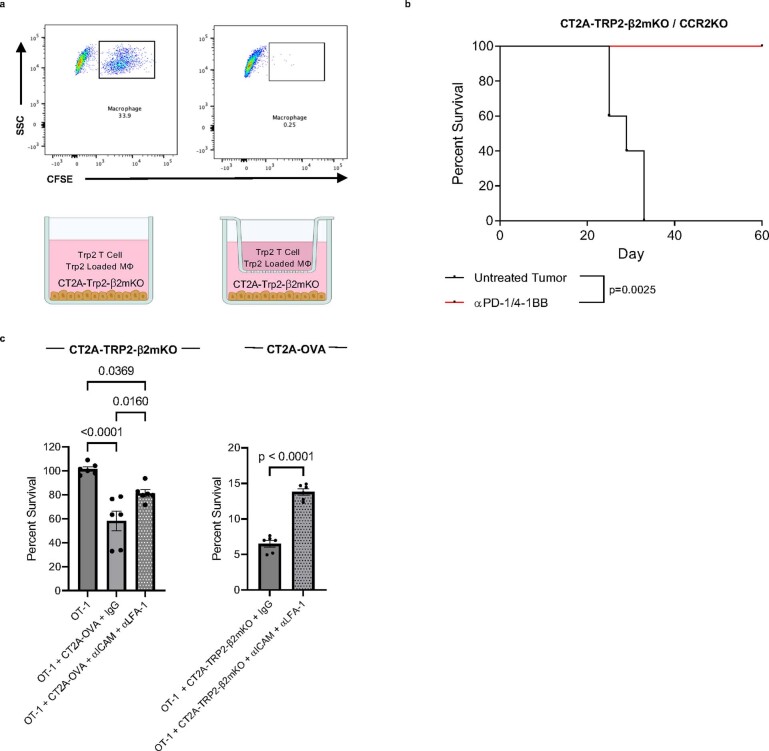
CD8^+^ T cell cytotoxicity against MHC-I-negative tumor cells is contact-dependent. **a**. CFSE-stained macrophages do not pass through 5.0μm Transwell™ membranes. TRP2 T cells, TRP2 Mϕ and CT2A-TRP2-β2mKO tumors were co-cultured either together or separated by a 5.0μm Transwell™ insert, as depicted. After 24 h of co-culture, the Transwell™ inserts were removed and the remaining cells were assessed with flow cytometry. **b**. Kaplan-Meier survival curve of CCR2^−^/^−^ mice implanted with CT2A-TRP2-β2mKO intracranially then treated with αPD-1/4-1BB (n = 5 mice/ group). Survival data were analyzed by two-sided Gehan-Breslow-Wilcoxon test. **c**. Percent survival of CT2A-TRP2-β2mKO or CT2A-TRP2 co-cultured with OT-1s alone, or combination with MHC-I-positive OVA positive glioma cells (CT2A-OVA) and blocking antibody for ICAM-1 and LFA-1 or IgG isotype control (n = 6 co-cultures/group). Experiments in **c** were repeated independently two times with similar results. P values were determined by two-tailed, unpaired Student’s t-test for **c** (left) and one-way ANOVA with post-hoc Tukey’s test for **c** (left). Data in **c** are shown as mean ± SEM. Source data

We then investigated whether macrophages or T cells more directly contributed to the contact-dependent tumor cytotoxicity mechanism. For these experiments, we instead used 5.0-μm Transwell plates, which allow for the smaller T cells, but not the larger macrophages, to pass through to the bottom of the well containing tumor cells (validation in Extended Data Fig. 3a). CT2A-TRP2-β2mKO tumor cells were placed on the opposite side of the Transwell insert from T cells and macrophages. This setup permitted T cells to first make direct contact with macrophages and subsequently pass through the membrane to make direct contact with tumor cells. The extent of tumor killing here was not significantly different from that in which all three cell types were in contact (Fig. 4a). This suggests that killing of MHC-I-negative tumors requires direct contact between tumor cells and TCR-stimulated T cells, while macrophages need only fill the role of providing antecedent antigen-specific T cell stimulation.

We now aimed to test whether such phenomena would persist in vivo. Earlier, we demonstrated that αPD-1/4-1BB immunotherapy was ineffective against i.c. CT2A-TRP2-β2mKO tumors implanted into OT-1 mice (Fig. 2e). In this model, all CD8^+^ T cells are specific for OVA, and TCR activation does not occur due to the absence of OVA antigen within the system. Our in vitro data above suggested that, although killing of MHC-I-negative tumors is tumor antigen agnostic, cognate antigen is still necessary for the required antecedent TCR activation. We therefore tested whether injecting cognate antigen-loaded macrophages into the tumor might prove sufficient for licensing an immunotherapeutic response against tumors lacking both MHC-I and the same cognate antigen. To accomplish this, we implanted MHC-I-negative, OVA-negative CT2A-TRP2-β2mKO gliomas orthotopically into OT-1 mice and subsequently administered cognate OVA-loaded macrophages or non-cognate TRP2-loaded macrophages into the tumor, followed by treatment with αPD-1/4-1BB immunotherapy (Fig. 4b). Tumor-bearing OT-1 mice implanted with OVA-pulsed macrophages demonstrated a significant survival advantage compared to OT-1 mice implanted with TRP2 macrophages (Fig. 4c). These data suggest that in vivo TCR activation is both necessary and sufficient to result in T cell-mediated killing of MHC-I-negative tumors in a tumor antigen-agnostic fashion.

We next sought to determine whether macrophages are exclusively required for TCR stimulation, or whether other MHC-I-positive APCs might also play that role. To assess this, we employed an anti-colony-stimulating factor 1 receptor (CSF1R) antibody to deplete macrophages in vivo before orthotopically implanting B16-OVA-β2mKO tumors into the flanks of mice^16^ (a subcutaneous melanoma model was used here to eliminate microglia as a confounding population). The depletion of macrophages had no effect on the growth of untreated tumors or on the effectiveness of αPD-1/4-1BB therapy (Fig. 4d). Similar findings were still encountered in the central nervous system, where the efficacy of αPD-1/4-1BB was maintained against CT2A-TRP2-β2mKO tumors implanted i.c. into *Ccr2*-KO mice, which have decreased tumor infiltration by peripheral macrophages^17^ (Extended Data Fig. 3b). In sum, these findings suggest that, while macrophages may indeed be sufficient for the adaptive priming stage of cytotoxicity against MHC-I-negative tumors, they are not specifically necessary.

To better assess other potential sources of the requisite TCR activation, we first performed in vitro cytotoxicity experiments by mixing CD8^+^ T cells with both MHC-I-positive and MHC-I-negative tumor cells, eliminating macrophages from the culture. Unsurprisingly, OVA-specific OT-1 T cells cultured only with MHC-I-negative, OVA-negative CT2A-TRP2-β2mKO tumor cells failed to result in tumor killing. However, the simple addition of MHC-I-positive OVA-positive (CT2A-OVA) tumor cells licensed T cell killing of antigen-mismatched CT2A-TRP2-β2mKO tumor cells (Fig. 4e). This suggested that antigen-cognate TCR activation by neighboring MHC-I-positive tumor cells might alone be sufficient to elicit CD8^+^ T cell killing of their MHC-I-negative counterparts. Likewise, simple TCR activation by activating anti-CD3 antibody also proved sufficient to induce killing of MHC-I-negative tumor cells in vitro (Fig. 4f). Interestingly, the addition of antibodies blocking lymphocyte function-associated antigen 1 (LFA-1) and intercellular adhesion molecule 1 (ICAM-1) modestly reduced in vitro cytotoxicity against both MHC-I-positive and MHC-I-negative tumors, providing further evidence of a T cell contact-dependent cytotoxicity mechanism (Extended Data Fig. 3c).

### NKG2D mediates T cell recognition of MHC-I-negative tumors

**Fig. 5:**
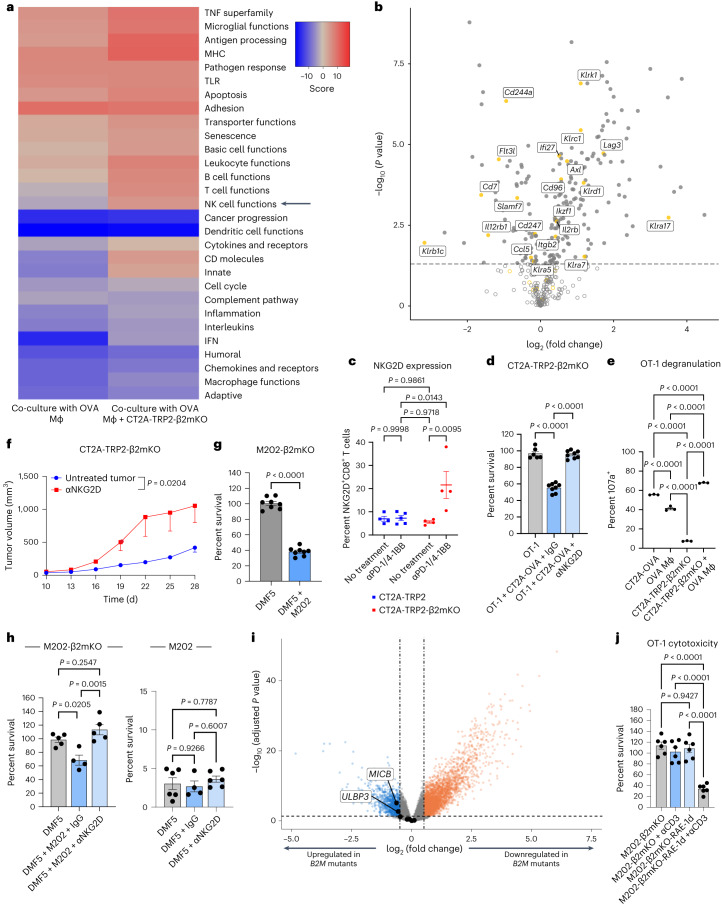
NKG2D on activated CD8^+^ T cells mediates killing of MHC-I-negative tumor cells in both murine and human tumor models. **a**, Gene set pathway analysis of RNA expression by OT-1 CD8^+^ T cells co-cultured with either OVA-pulsed macrophages alone or in conjunction with CT2A-TRP2-β2mKO cells, compared to OT-1 cells cultured alone (*n* = 3 co-cultures per group). Red, increased expression of pathway genes; blue, reduced expression. TLR, Toll-like receptor. **b**, Differential expression analysis of NK cell function genes between OT-1 cells cultured alone or co-cultured with OVA-pulsed macrophages and CT2A-TRP2-β2mKO cells. *P* values were adjusted for multiple testing (Methods). The horizontal dashed line is at an adjusted *P* value of 0.05. **c**, Percent of NKG2D^+^CD8^+^ T cells in tumors of mice bearing CT2A-TRP2 tumors treated with αPD-1/4-1BB (*n* = 5) or PBS (*n* = 4) or mice bearing CT2A-TRP2-β2mKO tumors and treated with αPD-1/4-1BB (*n* = 4) or PBS (*n* = 4). **d**, Percent survival of CT2A-TRP2-β2mKO cells co-cultured with OT-1 cells alone (*n* = 6 co-cultures) or in combination with CT2A-OVA glioma cells and either an immunoglobulin G (IgG) control (*n* = 8 co-cultures) or an NKG2D-blocking antibody (αNKG2D, *n* = 8 co-cultures). **e**, Percent of 107a^+^ OT-1 T cells co-cultured for 6 h with OVA-pulsed macrophages and MHC-I-positive or MHC-I-negative tumor cells assessed by flow cytometry (*n* = 3 co-cultures per group). **f**, Subcutaneous tumor growth of CT2A-TRP2-β2mKO tumors in C57BL/6 mice treated with either anti-NKG2D antibody or the PBS vehicle control (*n* = 8 per group). **g**, Survival of M202-β2mKO cells co-cultured with MART-1-specific DMF5 transduced T cells (DMF5) ± MART-1 stimulation provided by MHC-I-positive M202 tumor cells at a 1:1 effector:target ratio (*n* = 8 co-cultures per group). **h**, Survival of M202 cells in co-culture with either DMF5 T cells alone (*n* = 5 co-cultures) or with either the IgG isotype control (*n* = 4 co-cultures) or anti-NKG2D antibody (*n* = 5 co-cultures) (left) or M202-β2mKO cells (*n* = 6, *n* = 4, *n* = 6 co-cultures, respectively) (right) at a 1:1 effector:target ratio. **i**, Differential gene expression of PanCanAtlas (TCGA) samples with *B2M* mutations (*n* = 132) compared to 200 *B2M*-wild-type PanCanAtlas samples; dataset *P* values were adjusted for multiple testing (Methods). **j**, Percent survival of M202-β2mKO or M202-β2mKO-RAE-1d cells co-cultured with OT-1 cells with or without activating anti-CD3 antibody at a 1:1 effector:target ratio (*n* = 6 co-cultures per group). Growth curves in **f** were compared with two-way ANOVA. *P* values were determined using two-way ANOVA with Tukey’s multiple-comparison test for **c**, by one-way ANOVA with Tukey’s multiple-comparison test for **d**, **e**, **h** and **j** and by two-tailed, unpaired Student’s *t*-test for **g**. Experiments in **d**–**h** and **j** were repeated independently two times with similar results. Data in **c**–**h** and **j** are shown as mean ± s.e.m. Source data

**Extended Data Fig. 4:**
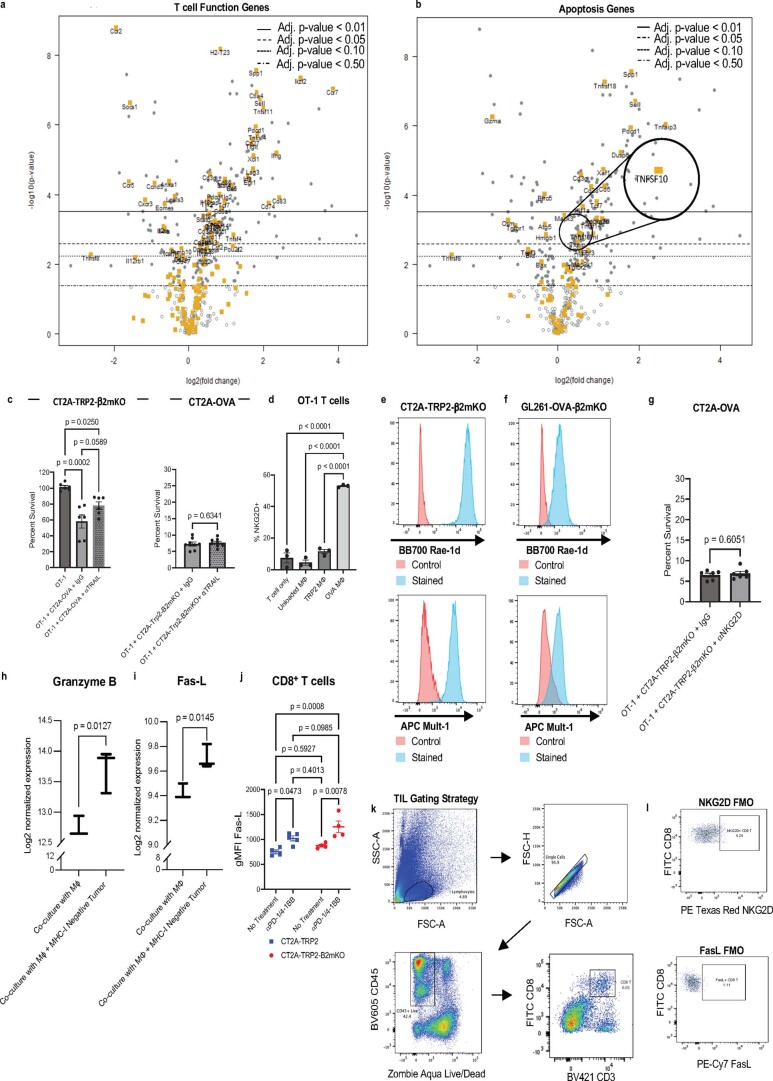
Characterization of MHC-I-independent cytotoxicity mechanism by CD8^+^ T cells. **a**–**b**. Differential expression analysis depicting log2 fold change versus log10 adjusted p value for differential expression of T cell function genes (**a**) or apoptotic function genes (**b**) between OT-1 T cells co-cultured with OVA macrophages and CT2A-TRP2-β2mKO tumors as compared with OT-1 T cells cultured alone (n = 3 co-cultures/group). P values adjusted for multiple testing (Methods). **c**. Survival of CT2A-TRP2-β2mKO cells or CT2A-TRP2 co-cultured with OT-1 CD8^+^ T cells and blocking antibody for TRAIL or isotype control 10:1 ET ratio (n = 6 co-cultures/group). **d**. Percent NKG2D^+^ OT-1 CD8^+^ T cells after 12 h in co-culture with antigen loaded Mϕ or alone in TCM + IL-2. 2:1 T cell to Mϕ ratio (n = 3 co-cultures/group). **e**–**f**. CT2A-TRP2-β2mKO (**e**) and GL261-OVA-β2mKO (**f**) expression of NKG2D ligands RAE-1d and MULT-1. **g**. Survival of CT2A-OVA in co-culture with OT-1 T cells, CT2A-TRP2-β2mKO tumors and IgG isotype control or αNKG2D 10:1 ET ratio (n = 6 co-cultures/group). **h**-**i**. Log2 Normalized expression of granzyme B (**h**) or Fas-L (**i**) in OT-1 T cells co-cultured with cognate antigen loaded macrophages or cognate antigen loaded macrophages and MHC-I-negative CT2A-TRP2-β2mKO (line at median with bars for min and max, n = 3 co-cultures/group). **j**. Geometric mean fluorescence intensity (gMFI) of Fas-L expression in tumor infiltrating CD8^+^ T cells from mice bearing CT2A-TRP2 tumors treated with αPD-1/4-1BB (n = 5) or PBS (n = 4) or mice bearing CT2A-TRP2-β2mKO and treated with αPD-1/4-1BB (n = 4) or PBS (n = 4). **k**. Gating strategy for tumor infiltrating lymphocytes in **j** and Figs. 2a & 5c. **l**. Gating for NKG2D^+^ and FasL^+^ CD8^+^ T cell in Fig. 5c and Extended Data Fig. 4j, respectively. P values were determined using two-way ANOVA with post-hoc Tukey’s test for **j**, one-way ANOVA with post-hoc Tukey’s test for **c** (left) & **d**, and by two-tailed, unpaired Student’s t-test for **c** (right) **g**-**i**, **l**. Data in **c**,**d**,**j & g** are shown as mean ± SEM. In vitro data are representative findings from one of a minimum of two independently repeated experiments with similar results. ET ratio, effector to target ratio. FMO fluorescence minus one. TIL: Tumor infiltrating lymphocyte. Source data

In an effort to clarify the nature of the cell–cell contact-dependent mechanism underlying CD8^+^ T cell recognition of MHC-I-negative tumors, we first employed an unbiased approach to examine differences in inflammatory gene expression profiles in antigen-specific CD8^+^ T cells exposed to both MHC-I-negative tumors and cognate antigen-loaded macrophages. Gene sets from primed OT-1 T cells co-cultured with OVA-pulsed macrophages either alone or in combination with antigen-negative CT2A-TRP2-β2mKO tumor cells were both compared to primed OT-1 T cells alone. OVA-negative β2mKO tumor lines were used to ensure the absence of tumor–TCR interactions. OT-1 TCR activation for these experiments was accomplished by concomitant culture with OVA-pulsed macrophages. TCR-activated OT-1 cells exposed to CT2A-TRP2-β2mKO cells exhibited increased expression of genes denoting both NK and T cell functions, increases not seen simply with TCR activation of OT-1 cells (Fig. 5a). Within the T cell function gene set, differentially expressed genes included those encoding activation markers but not receptors involved in direct contact-mediated cytotoxicity (Extended Data Fig. 4a). Notably, in the apoptosis gene set, expression of *Tnfsf10*, which encodes the TNF-related apoptosis-inducing ligand (TRAIL), was slightly increased (log_2_ (fold change) = 0.562, adjusted *P* value = 0.0262) (Extended Data Fig. 4b). Therefore, given its known role in contact-dependent T cell cytotoxicity, we investigated whether TRAIL might be involved in killing MHC-I-negative tumors in vitro. However, blocking TRAIL did not significantly reduce T cell-mediated in vitro cytotoxicity directed against either MHC-I-positive or MHC-I-negative tumors (Extended Data Fig. 4c).

Within the NK cell function gene set, expression of *Klrk1*, which encodes the cytotoxic receptor NKG2D, was especially prominent in OT-1 cells exposed to CT2A-TRP2-β2mKO cells (Fig. 5b, log_2_ (fold change) = 1.1, adjusted *P* value = 3.25 × 10^−5^). NKG2D is an activating receptor traditionally associated with cytotoxic function in NK cells, although it is also expressed on activated CD8^+^ T cells for which its role has mainly been described as co-stimulatory^18–21^. In mice, the ligands for NKG2D are the β2m-independent non-classical MHC-I molecules RAE-1, H60 and MULT-1 (ref. ^18^). Unlike classical MHC-I, which is expressed on all nucleated cells, non-classical MHC-I expression is generally induced by stress and is upregulated on tumor cells^20,22,23^. The expression of non-classical MHC-I on tumor cells has been shown to be further upregulated following exposure to chemotherapy or ionizing radiation^24^.

Similarly, following TCR activation, engagement of the NKG2D receptor by its ligands on a target cell activates cytotoxic effector functions in the NKG2D^+^ cell, including the release of cytotoxic granules and expression of Fas ligand (FasL)^25–29^. NKG2D effector functions in CD8^+^ T cells, specifically, appear to be dependent on concurrent TCR activation to limit effector activity to appropriate surrounding targets^27,30^. In murine CD8^+^ T cells, NKG2D expression is upregulated in response to TCR activation (Extended Data Fig. 4d). As CD8^+^ T cell-mediated killing of MHC-I-negative tumors in our models was likewise dependent on TCR activation but did not require antigen-cognate interactions between T cells and tumor cells, we hypothesized that NKG2D might be an important contributor to the tumoricidal mechanism. To initially assess this, we began by examining NKG2D expression on T cells in our models. Accordingly, we examined differences in NKG2D expression on CD8^+^ T cells infiltrating either CT2A-TRP2 or CT2A-TRP2-β2mKO gliomas in untreated mice or in mice treated with αPD-1/4-1BB. NKG2D levels proved significantly higher on CD8^+^ T cells infiltrating treated MHC-I-negative CT2A-TRP2-β2mKO tumors than on either treated MHC-I-positive tumors or untreated tumors (Fig. 5c). Detecting such increased levels of NKG2D on T cells, we conversely examined two of our MHC-I-negative tumor lines for the presence of the relevant NKG2DLs. The CT2A-TRP2-β2mKO line was found to highly express the non-classical MHC-I molecules RAE-1 and MULT-1, findings that were recapitulated with the additional glioma line GL261-OVA-β2mKO (Extended Data Fig. 4e,f).

To examine the functional contribution of NKG2D to CD8^+^ T cell killing of MHC-I-negative tumors, we repeated our prior in vitro tumor cytotoxicity studies, this time in the presence or absence of NKG2D-blocking antibody. OT-1 T cells were again cultured with OVA-negative, MHC-I-negative CT2A-TRP2-β2mKO tumor cells, although, for these experiments, we used CT2A-OVA cells as the source of OVA antigen presentation and OT-1 TCR activation. As with our previous results, OT-1 T cells successfully killed CT2A-TRP2-β2mKO tumors, but only when an OVA-presenting cell was likewise present (Fig. 5d). Killing of CT2A-TRP2-β2mKO cells, however, was completely abrogated in the presence of anti-NKG2D-blocking antibody (Fig. 5d). The use of a CT2A-OVA tumor as a source of OVA TCR stimulation also permitted us to evaluate the impact of anti-NKG2D therapy on concomitant killing of MHC-I-positive tumor cells. No such impact was seen, as CT2A-OVA cells were readily killed under either condition (Extended Data Fig. 4g).

**Extended Data Fig. 5:**
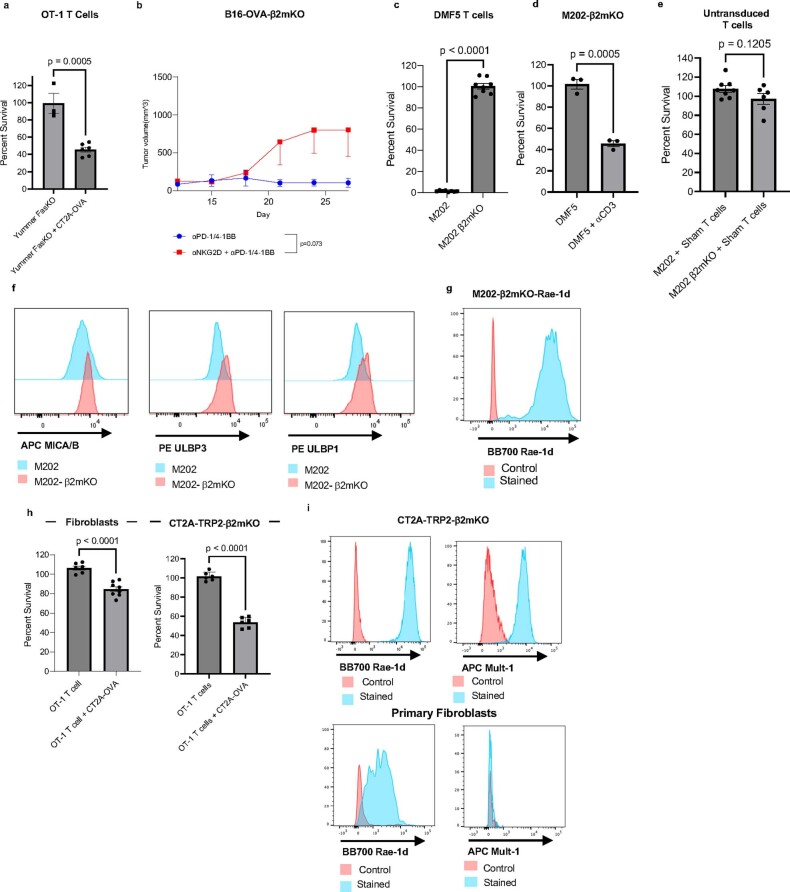
NKG2D-mediated killing of MHC-I-negative tumors is recapitulated by human CD8 + T cells and is limited in healthy tissues. **a**. Survival of Yummer FasKO cells co-cultured with OT-1 T cells (n = 3 co-cultures/group), or OT-1 T cells and OVA+ tumor 10:1 ET ratio (n = 6 co-cultures/group). **b**. Subcutaneous tumor growth curve of B16-OVA-β2mKO, treated with either αPD-1/4-1BB or αNKG2D and αPD-1/4-1BB, n = 8 mice/group). **c**. Percent survival of MART-1 antigen positive M202 or M202-β2mKO melanoma cells co-cultured with MART-1 specific DMF5 transduced T cells (DMF5). 1:1 ET ratio (n = 8 co-cultures/group). **d**. Survival of M202-β2mKO cells co-cultured with DMF5 T cells +/− activating human αCD3 antibody. 1:1 ET ratio (n = 3 co-cultures/group). **e**. Survival of M202 (n = 8 co-cultures/group) or M202-β2mKO (n = 6 co-cultures/group) after 24 h with untransduced human T cells (Sham T) 1:1 ET ratio. **f**. M202 and M202-β2mKO expression of human NKG2D ligands MIC-A / MIC-B, ULBP3 and ULBP1. **g**. M202-β2mKO tumors were engineered to express murine NKG2D ligand RAE-1d. **h**. Survival of primary mouse dermal fibroblast cells or CT2A-TRP2-β2mKO cells co-cultured with OT-1 T cells (n = 6 co-cultures/group) and OVA+ tumor (CT2A-OVA) (n = 8 co-cultures/group), 10:1 ET ratio. **i**. Primary fibroblast and CT2A-TRP2-β2 mKO expression of NKG2D ligands RAE-1d and MULT-1. Tumor growth curves in **b** were compared with two-way ANOVA. p values were determined by two-tailed, unpaired Student’s t-test for **a**, **c**–**e**, & **h**. Data in **a,b**, **c**–**e**, & **h** are shown **as** mean ± SEM. Experiments in **a-i** were repeated independently two times with similar results. ET ratio, effector to target ratio. Source data

The downstream cytotoxic effectors of NKG2D signaling can include granzymes, perforin and FasL^29,31^, raising the question as to which of these might be mediating MHC-I-negative tumor cell killing in our system. Our expression analysis data revealed an increase in granzyme B and FasL expression (Extended Data Fig. 4h,i) in TCR-activated T cells exposed to MHC-I-negative tumors, suggesting that degranulation may play a role in the tumoricidal mechanism. Indeed, degranulation by OT-1 T cells was increased in the presence of MHC-I-negative tumor cells and cognate antigen-loaded macrophages as compared with that with macrophages alone (Fig. 5e). Given the results of prior studies^32–34^, we additionally specifically tested the role of FasL–Fas interactions. FasL expression on tumor-infiltrating CD8^+^ T cells was increased in response to immunotherapy but was not significantly different across CD8^+^ T cells isolated from MHC-I-positive versus MHC-I-negative tumors (Extended Data Fig. 4j–l). Additionally, in vitro, antigen-negative Fas receptor-KO (FasKO) melanomas were still susceptible to killing by TCR-activated CD8^+^ T cells (Extended Data Fig. 5a), suggesting that FasL–Fas interactions are not critical for the tumoricidal mechanism.

To determine whether blocking NKG2D might similarly abrogate killing of MHC-I-negative tumors in vivo, we subcutaneously implanted CT2A-TRP2-β2mKO tumors into C57BL/6 mice and treated them with anti-NKG2D antibody or a vehicle control. Mice treated with anti-NKG2D antibody exhibited a significantly increased rate of tumor growth, akin to that seen in our in vitro models (Fig. 5f). To additionally assess the impact of NKG2D blockade on a more potent mode of tumor killing involving immunotherapy, we used ICB-responsive B16-F10 melanomas treated concomitantly with αPD-1/4-1BB. Administering anti-NKG2D antibody reduced the efficacy (although not significantly, *P* = 0.073) of αPD-1/4-1BB in these tumors, increasing the rate of tumor growth (Extended Data Fig. 5b).

Having examined the role of NKG2D both in vitro and in vivo in mice, the question remained as to whether similar mechanisms for killing of MHC-I-negative tumors might be at play in human cancers. To address the translational relevance, we performed in vitro cytotoxicity experiments using the M202 human melanoma line (human leukocyte antigen (HLA)-A*0201), which expresses the MART-1 tumor-associated antigen, or an MHC-I-negative *B2M*-KO version of the same cells (M202-β2mKO). Tumors were co-cultured with DMF5 TCR-transduced human T cells (DMF5 T cells), which recognize MART-1 presented in the context of HLA-A*0201 (refs. ^35–37^). Similar to our murine experiments, DMF5 T cells alone efficiently killed MHC-I-positive M202 cells but failed to kill M202-β2mKO cells unless undergoing concurrent MART-1 TCR stimulation or non-specific TCR stimulation with anti-CD3 antibody (Fig. 5g and Extended Data Fig. 5c,d). Sham-transduced human CD8^+^ T cells failed to kill either M202 or M202-β2mKO cells in vitro (Extended Data Fig. 5e). Furthermore, the addition of anti-NKG2D antibody entirely abrogated killing of MHC-I-negative M202-β2mKO cells but had no effect on the killing efficiency of MHC-I-positive M202 cells (Fig. 5h). These data suggest that NKG2D-mediated T cell killing of MHC-I-negative tumors is pertinent to and persists amid human cancer.

We next stained M202-β2mKO cells for expression of human NKG2DLs and found that they expressed high levels of MICA, MICB, ULBP3 and ULBP1, with each being more highly expressed on M202-β2mKO cells than on parental MHC-I-positive M202 cells (Extended Data Fig. 5f). We therefore assessed NKG2DL expression in human β2m-mutant tumors using The Cancer Genome Atlas (TCGA) PanCanAtlas dataset. Differential expression analysis demonstrated significantly higher expression of NKG2DLs MICB and ULBP3 in *B2M* mutants than in human *B2M*-wild-type tumors (Fig. 5i). Higher expression of NKG2DLs MICA and MICB was also recently demonstrated in the setting of *B2M*-mutant mismatch repair-deficient colon, stomach and uterine cancers^38^. Thus, MHC-loss variant tumor cells would appear to be more susceptible to NKG2D-mediated recognition.

The question remained, then, as to whether simply furnishing a cell with NKG2DL would be sufficient to mediate its MHC-I-independent killing by CD8^+^ T cells. To assess this, we engineered a human *B2M*-KO melanoma line (M202-β2mKO) to express the mouse NKG2DL RAE-1d (Extended Data Fig. 5g). Human tumor cells were used, as there is no baseline level of murine NKG2DL expression. Allogeneic reactivity was rendered irrelevant by the absence of MHC-I (Fig. 5j). As expected, even following TCR activation by anti-CD3 therapy, murine OT-1 T cells remained unable to kill untransfected MHC-I-negative human cells (Fig. 5j). Notably, however, murine OT-1 T cells readily killed human M202-β2mKO melanoma cells engineered to express murine RAE-1d (Fig. 5j). These data, in turn, raised the question as to how tumor specificity might be maintained in the microenvironment, if NKG2DL expression is sufficient to mediate antigen-indiscriminate killing. To further evaluate risk to non-tumor cells, we performed cytotoxicity experiments using primary mouse fibroblasts (exposed to MHC-replete tumor cells to simulate the tumor environment) and observed these cells to be significantly less susceptible to killing than MHC-I-negative tumor cells placed under the same conditions (Extended Data Fig. 5h). Accordingly, primary fibroblasts were found to also express significantly lower levels of RAE-1d than CT2A-TRP2-β2mKO cells and had no expression of MULT-1 (Extended Data Fig. 5i). These data suggest that the higher levels of NKGD2L observed on β2m-mutant and β2mKO tumors make them more susceptible to NKG2D-mediated CD8^+^ T cell killing, while lower NKGD2L levels on surrounding normal tissues may be protective against this antigen-indiscriminate cytotoxicity mechanism.

## Discussion

Traditional models of anti-tumor immunity have centered on tumor cell killing as a function of CD8^+^ T cell recognition of cognate antigen presented exclusively in the context of tumor cell MHC-I molecules^1,2,9,39^. Here, we report an alternate T cell mechanism for tumor cytotoxicity that depends neither on tumor antigen nor on tumor MHC-I expression. This mechanism is particularly active and effective against MHC-I-loss variants and is mediated instead by T cell NKG2D engagement of non-classical MHC-I (NKG2DLs) on tumor cells in an antigen-independent and indiscriminate fashion. Subsequent tumor cell killing depends on prior TCR-mediated T cell activation (even if by an ultimately tumor-irrelevant antigen), meaning that adaptive priming is then paired with subsequent innate killing^40^. Cell killing itself appears to involve cytotoxic degranulation with granzyme and perforin. This mechanism remains active in tumors containing a mixture of MHC-I-replete and MHC-I-deficient cells, where TCR activation can seemingly be provided by either MHC-I-replete tumor cells or adjacent myeloid APCs. Likewise, the mechanism extrapolates to human tumors and T cells, suggesting clinically relevant findings.

Our findings call into question the conventional notion that tumor downregulation of MHC-I necessarily represents an effective mode of immune escape^41–43^. Results from recent clinical studies appear to corroborate that such notions may indeed be faulty^11,44–48^. Within glioblastoma alone, for instance, two separate studies gathering single-cell RNA sequencing data from human tumors have found *B2M* expression to be inversely correlated with survival^11,48^. Likewise, additional studies have implied that MHC-I loss does not necessarily render tumor cells invulnerable to CD8^+^ T cell-dependent immunotherapies, including ICB^10,49^. The perception of MHC-I loss as a means of cancer immune escape, then, is perhaps best described as controversial.

While some studies to date have indeed highlighted a possible prognostic benefit to tumor MHC-I loss, these have often attributed such benefit to a sudden conferred susceptibility to NK cell-mediated killing^10,44,47^. Strikingly, however, we demonstrate that NK cells are not required for the efficacy of an ICB-involving regimen employed against MHC-I-negative tumors. Additionally, while recent studies have found γδ T cells to be effectors against MHC-I-negative tumors, we have shown that this function extends to CD8^+^ T cells, a particular surprise given the previously held requirements of CD8^+^ T cell-mediated killing for MHC-I^38^. By stark contrast, we find that CD8^+^ T cells are indeed required to maintain immunotherapeutic efficacy against MHC-I-negative tumors, despite the absence of a traditional T cell killing mechanism.

An interesting facet of our data is the apparent antigen independence of the described T cell killing mechanism. While CD8^+^ T cell cytotoxicity in our studies required relatively concurrent TCR activation, subsequent TCR-mediated recognition of tumor cells was not required. Therefore, tumor cells lacking MHC-I need not be present or even express the same antigen that was enlisted for T cell activation. Instead, only tumor expression of NKG2DL was required (and sufficient), and tumor cells lacking MHC-I appeared to have increased expression of these ligands, leaving them more susceptible to this mode of cell killing. The implication, however, is that T cells specific for irrelevant antigens need only be activated in the vicinity of tumors lacking MHC-I but possessing NKG2DLs to effect a cytotoxic response. In this manner, polyclonally or agnostically activated T cells can become potential effectors in this cytotoxicity model.

As target NKG2DL expression proved sufficient to mediate T cell killing, the concern arises for how tumor specificity (and self-tolerance) might be maintained within the TME in the face of antigen-indiscriminate cytotoxicity. The requirements for relatively concomitant adaptive T cell priming, higher expression of NKG2DL on MHC-I-negative tumors and relatively low levels of NKG2DL on neighboring healthy cells all may provide a modicum of tumor selectivity and protection. Additionally, and interestingly, CD8^+^ T cells co-cultured with MHC-I-negative tumor cells and cognate antigen-loaded macrophages upregulated the inhibitory receptor NKG2A (*Klrc1*) (Fig. 5b), which binds to β2m-dependent Qa-1 (HLA-E in humans)^50,51^. While Qdm or Qa-1 is not present in our β2mKO tumor models, concurrent upregulation of inhibitory receptors on T cells may further constrain this mechanism from affecting healthy non-tumor cells within the TME, should they express the relevant ligands.

These findings, while surprising, are perhaps not entirely without precedent. While the role of NKG2D in CD8^+^ T cells has mainly been described as co-stimulatory^18–21^, it has previously been shown that NKG2D receptor–ligand interactions can lead to the formation of immunologic synapses between T cells and target cells in an antigen-independent manner^19^. Our findings of a contact-dependent cytotoxicity mechanism with evidence of degranulation support this notion of immunologic synapse formation by NKG2D in an antigen-independent manner.

Interestingly, Upadhyay et al. recently described a bystander killing phenomenon by chimeric antigen receptor T cells in the context of mixed antigen-positive and antigen-negative tumors^32^. While chimeric antigen receptor T cells act in an MHC-independent fashion regardless, the authors also reported the ability of endogenous bystander T cells to kill a mixed MHC-I-positive and MHC-I-negative tumor in vitro, in a seemingly Fas-dependent fashion. Direct activation of the TCR was required and was provided by MHC-replete tumor cells. While we uncovered a similar phenomenon, we also show that TCR stimulation can come from non-tumor sources (that is, myeloid cells) and is thus sufficient to mediate killing of tumors entirely devoid of MHC-I. Our findings are further contrasted, as we additionally highlight a role for NKG2D in mediating tumor cell recognition and killing, a mechanism that persists when Fas is absent. We instead suggest a role for perforin and granzyme in mediating tumor cell killing following NKG2D-based tumor recognition.

Here, we characterize an MHC-I- and antigen-independent CD8^+^ T cell mechanism for tumor cell killing that is seen consistently in vivo as well as in human tumor cells. We demonstrate that CD8^+^ T cell-dependent immunotherapies can indeed remain effective against tumors, even when uniformly lacking MHC-I. Further investigations into the role of NKG2D and NKG2DLs in mediating tumor immunotherapeutic susceptibility as well as into other potential therapeutic implications for these findings are warranted.

## Methods

### Ethics statement

The research performed in this study complies with all ethical regulations. All mouse experiments were approved by the Institutional Animal Care and Use Committee (IACUC) at Duke University Medical Center (protocol A163-21-08). The maximum subcutaneous tumor size permitted by the Duke University Medical Center IACUC is 2,000 mm^3^. This maximum tumor size was never exceeded in the studies. Source data are provided for all in vivo experiments.

### Mice

The IACUC at Duke University Medical Center approved all experimental procedures. Animal experiments involved the use of female mice at 6–12 weeks of age. Animals were maintained under pathogen-free conditions, in temperature- and humidity-controlled housing, with free access to food and water, under a 12-h light–dark cycle at the Cancer Center Isolation Facility of Duke University Medical Center. All experimental procedures were approved by the Institutional Animal Care and Use Committee at Duke University Medical Center. C57BL/6 mice were purchased from Charles River Laboratories. OT-1 (003831), CD8KO (002665) and *Ccr2*-KO (004999) mice were purchased from Jackson Laboratories. Mice were housed at the Duke University Medical Center Cancer Center Isolation Facility under pathogen-free conditions. Female mice were used for in vivo survival experiments to avoid unwanted immunogenicity related to sex chromosomes. Sex was not considered in the study design.

### Cell lines

Murine cell lines included CT2A glioma, GL261 glioma, B16-F10 (B16) melanoma and YUMMER-FasKO melanoma lines. The YUMMER-FasKO cell lines were kindly provided by K. Wood (Duke University). All murine cell lines are syngeneic in C57BL/6 mice. We transfected CT2A cells to express the antigen TRP2 to generate CT2A-TRP2 cells. Additionally, we transfected GL261 and B16 cells to express OVA to generate GL261-OVA and B16-OVA cells, respectively. To KO *B2m* in CT2A-TRP2, GL261-OVA and B16-OVA cells, we used a CRISPR-based strategy and the LentiCRISPRv2 plasmid (52961, Addgene) as described previously^52^. Briefly, the lentiCRISPRv2 vector was digested with BsmBI (NEB), purified by gel electrophoresis using the QIAquick Gel Extraction Kit (Qiagen), ligated with phosphorylated oligonucleotides and transformed into Stbl3 bacteria. Positives clones were verified and sequenced. We used gRNA that targets the first exon of the *B2m* gene, and the sequence is as follows: 5′-CCGAGCCCAAGACCGTCTAC. HEK293T cells (kindly provided by J. Sampson, Duke University) were then transfected with LentiCRISPRv2-β2m, psPAX2 (Addgene, 12260) and pVSVg (Addgene, 8454). Transfected cells were cultured for 48 h, and then supernatant was collected, and tumor cells were transduced with Polybrene Infection/Transfection Reagent (Sigma-Aldrich) at 8 µg ml^−1^. Cells were cultured in viral supernatant for 24 h. The medium was changed, and cells were cultured for another 5 d. H2K^b^- and H2D^b^-negative cells were single-cell sorted and confirmed by flow cytometry. C57BL/6 murine adult primary dermal fibroblast cells were obtained from Cell Biologics (C57-6067). The human melanoma M202 and M202-β2mKO cell lines were kindly provided by A. Ribas (UCLA). All cell lines with the exception of YUMMER were cultured in vitro in Dulbecco’s Modified Eagle Medium (DMEM) with 2 mM l-glutamine and 4.5 mg ml^−1^ glucose (Gibco) containing 10% FBS. YUMMER cells were cultured in DMEM/F12 containing 10% FBS and supplemented with non-essential amino acids. To generate M202-β2mKO cells expressing murine RAE-1d knockin, cDNA encoding mouse RAE-1d was cloned into the retroviral MSGV1 backbone. To generate retrovirus, HEK293T packaging cells were co-transfected with plasmids encoding mRAE-1d, Gag–Pol and VSV-G. Retrovirus was collected 48 h later and used to transduce M202-β2mKO human melanoma cells at a concentration of 1 ml viral supernatant per 10^6^ M202-β2mKO cells. Three days later, M202-β2mKO cells expressing high levels of murine RAE-1d were sorted by flow cytometry. M202-β2mKO-RAE-1d cells were expanded before use in subsequent experiments. All cell lines were authenticated and tested negative for mycoplasma and interspecies contamination by IDEXX Laboratories.

### In vivo tumor experiments

Tumor cells were collected in their logarithmic growth phase via trypsinization (Gibco, 25300-054) and then resuspended in PBS. For i.c. implantation, tumor cells were mixed 1:1 with 3% methylcellulose and loaded into a 250-µl Hamilton syringe (Hamilton, 81120). Mice were anesthetized using isoflurane. Injection sites were shaved, and then mice were placed in a stereotactic frame. After sterilization of the scalp, a midline incision was made to expose the bregma. The Hamilton syringe was positioned over the bregma, moved 2 mm laterally to the right, lowered 5 mm below the surface of the skull and then raised 1 mm to create a pocket for the tumor suspension.

An infusion pump was then used to infuse 5 µl of tumor cells at 120 µl min^−1^. For CT2A-TRP2, 2.5 × 10^4^ cells were injected; for CT2A-TRP2-β2mKO, 5.0 × 10^4^ cells were injected. These doses were determined by tumorgenicity experiments performed to determine the dose required for a median survival of around 21 d. After completing the infusion, the syringe was left in place for an additional 45 s before removal. Bone wax was used to cover the injection site, and then the incision was stapled close. Mice were euthanized if there was any bulging of the skull or eyes or if they experienced failure to ambulate.

For subcutaneous implantation, tumor cells were collected as described above and resuspended in PBS. The appropriate numbers of tumor cells were administered in 200 μl PBS under the skin of the right flank. For B16-OVA-β2mKO, 2.5 × 10^5^ cells were injected subcutaneously. For CT2A-TRP2-β2mKO, 1 × 10^6^ cells were injected subcutaneously. These doses were determined by tumorgenicity experiments. Tumors were measured every 3 d. A maximal tumor burden of 2,000 mm^3^ was not exceeded. Mice were killed when tumor volume reached 2,000 mm^3^, tumors were over 20 mm in one direction, or tumors became ulcerated. Following killing, tumors were subsequently assigned the endpoint target volume of 2,000 mm^3^.

Tumors were implanted into the indicated mouse strain for each experiment. Mice were randomly assigned to treatment groups within a given genotype. Humane endpoint checks for mice were performed by an animal technician blinded to expected outcomes. In adoptive transfer experiments with TRP2-specific T cells, we administered 10^7^ TRP2 TCR-transduced T cells intravenously 7 d after tumor implantation. For adoptive transfer experiments with antigen-loaded macrophages, we pulsed macrophages with the OVA_257–264_ (SIINFEKL) peptide and then administered 5 × 10^5^ macrophages i.c. at the tumor site 5 d after tumor implantation. Unless otherwise indicated, mice were monitored for survival or killed once they reached a humane endpoint.

### In vivo antibody treatment

Antibodies for in vivo treatment were obtained from BioXCell. These include anti-mouse PD-1 (RMP1-14, BE0146), anti-mouse 4-1BB (LOB12.3, BE0169), anti-mouse CD8α (2.43, BE0061), anti-mouse CD4 (GK1.5, BE0003-1), anti-mouse NK1.1 (PK136, BE0036), anti-mouse NKG2D (HMG2D, BE0111) and anti-mouse CSF1R (AFS98, BE0213) antibodies. For in vivo treatment with anti-PD-1 and anti-4-1BB antibodies, 200 µg each of anti-PD-1 and anti-4-1BB antibodies was diluted with PBS for a total injection volume of 200 µl. Mice received intraperitoneal (i.p.) injections of ICB (anti-PD-1 and anti-4-1BB antibodies) every 3 d, starting on day 9 after tumor implantation, for a total of four treatments, unless otherwise specified. For antibody depletion of CD8^+^ T cells, CD4^+^ T cells and NK cells, 200 µg of anti-CD8α, anti-CD4 or anti-NK1.1 antibody was diluted with PBS for a total injection volume of 200 µl. For depletions beginning before tumor implantations, mice received i.p. injections each day for 3 d, with the last dose given 1 d before tumor implantation. Maintenance doses were then given every 6 d. Control treatments consisted of 200 µl PBS. Depletion of CD8^+^ (BioLegend APC anti-mouse CD8a clone QA17A07, 155005), NK (BioLegend AF488 NK1.1 clone PK136, 108718; BioLegend PE NKp46, 137611) and CD4^+^ (BioLegend PE CD4 clone GK1.5, 100408) cells was validated by flow cytometry of blood samples before tumor implantation and again in the blood of tumor-bearing mice 1 d before the scheduled maintenance dose. For in vivo blockade of NKG2D, 250 µg of anti-NKG2D antibody was given starting at day 8, continuing every 3 d until humane endpoints were reached^53^. For in vivo depletion of macrophages, 400 µg of anti-CSF1R antibody was diluted in PBS for a total injection volume of 200 µl. Antibody depletion was started 3 d before tumor implantation, and maintenance doses were then given every 3 d for the duration of the experiment^16^.

### Generation of antigen-specific T cells

We engineered TRP2 TCR T cells by retroviral transduction of T cells with the pMX-TRP2-TCRβ-2A-α vector (a kind gift from T. Schumacher, Netherlands Cancer Institute) as described previously^54^. This TCR recognizes the TRP2_180–188_ epitope in the context of H2K^b^. Briefly, we transfected HEK293T cells with vectors encoding the TRP2 TCR and retroviral packaging genes to generate TCR-encoding retrovirus. TRP2 TCR retrovirus was then used to transduce mouse T cells 48 h after activation with concanavalin A in the presence of 50 U ml^−1^ human IL-2. Transduction was performed in non-tissue culture 24-well plates previously coated with 0.5 ml RetroNectin (Takara) at a concentration of 25 μg ml^−1^ in PBS. Cells were split every 48 h for 5 d in mouse T cell medium (RPMI with 10% FBS, non-essential amino acids, l-glutamine, sodium pyruvate, β-mercaptoethanol, penicillin–streptomycin and gentamycin) before use.

We engineered human T cells to express the DMF5 TCR that recognizes the MART-1 antigen expressed by the human melanoma M202 cell line^8,35,36^. DMF5 TCR-α and TCR-β were cloned into the MSGV1 retroviral backbone as a bicistronic message with a P2A self-cleaving peptide separating the α and β domains. DMF5 TCR retrovirus was generated by transfecting HEK293T cells with the DMF5 TCR MSGV1 vector and separate vectors encoding Gag–Pol and the gibbon–ape leukemia virus envelope. Anonymous healthy donor peripheral blood mononuclear cells (Stemcell) were activated with anti-CD3 antibody (OKT3, BioLegend) in the presence of 100 U ml^−1^ human IL-2. After 48 h, activated T cells were transduced with DMF5 TCR retrovirus to generate DMF5 T cells. DMF5 T cells were split into fresh human T cell medium (RPMI supplemented with 10% FBS, l-glutamine, HEPES, sodium pyruvate, non-essential amino acids and penicillin–streptomycin) plus 100 U ml^−1^ IL-2 every 48 h.

OT-1 T cells were isolated from OT-1 mice by culturing OT-1 splenocytes in T cell medium supplemented with 50 IU ml^−1^ IL-2 and 1 μM OVA SIINFEKL peptide (AnaSpec) for 48 h. Cells were purified for CD8^+^ T cells as described above and subsequently cultured in TCM with 50 IU ml^−1^ IL-2, splitting every 24 h for a total of 4 d.

### Monocyte purification, macrophage differentiation and antigen loading

Ly6C^hi^ monocytes were purified from bone marrow as previously described^55^. Briefly, bone marrow was flushed from the tibiae, femora, humeri and sternum of C57BL/6, CD45.1 (B6.SJL-*Ptprc*^*a*^ *Pepc*^*b*^/BoyJ) or β2mKO (B6.129P2-*Β2mKO*^*tm1Unc*^/DcrJ, Jackson Laboratory stock 002087) mice into cRPMI-10 medium (glutamine-free RPMI-1640 medium with 10% FBS, 100 U ml^−1^ penicillin, 100 µg ml^−1^ streptomycin, 100 µM MEM non-essential amino acids, 2 mM l-glutamine and 1 mM sodium pyruvate). Red blood cells (RBCs) were lysed with ammonium–chloride–potassium buffer at room temperature for 2 min and neutralized with cRPMI-10. The cell suspension was then passed through a 70-µm nylon cell strainer and incubated for 30 min at 4 °C in separation buffer (0.5% bovine serum albumin, 2 mM EDTA in PBS) containing biotinylated anti-CD3ε, anti-CD4, anti-CD8α, anti-CD11c, anti-CD19, anti-B220, anti-CD49b, anti-I-A^b^, anti Sca-1, anti-c-Kit, anti-TER-119 and FITC-conjugated anti-Ly6G and anti-CCR3 (5 μl ml^−1^ for anti-CCR3 antibody; all others at 1.25 μl ml^−1^) antibodies. Cells were then washed with labeling buffer (2 mM EDTA in PBS) and then incubated for 15 min at 4 °C in labeling buffer containing streptavidin-conjugated and anti-FITC MicroBeads (Miltenyi Biotec). Cells were negatively selected using MACS LD columns according to the manufacturer’s instructions. The resulting classical monocytes (>90% purity) were cultured for 5–7 d in non-tissue culture-treated dishes containing macrophage differentiation medium (glutamine-free RPMI-1640 medium with 20% FBS, 100 U ml^−1^ penicillin, 100 µg ml^−1^ streptomycin, 100 µM MEM non-essential amino acids, 2 mM l-glutamine, 1 mM sodium pyruvate and 50 ng ml^−1^ recombinant murine M-CSF (PeproTech, 315-02)). To prevent macrophage polarization, no additional cytokines were added. Monocyte-derived macrophages were loaded with antigen by adding 10 µM peptide dissolved in DMSO to the culture for 4 h or an equal volume of DMSO vehicle-only control. BMDMs were then washed twice with PBS to remove remaining antigen, separated from culture plates using a non-enzymatic dissociation agent (Corning Cellstripper, 25-056-Cl) and counted.

### Tissue processing and flow cytometry

A detailed protocol for processing and staining tumors can be found in ref. ^56^. In brief, after perfusion with PBS and 1% heparin, tumor-bearing hemispheres were collected on day 18 after tumor implantation. A single-cell suspension was generated and passed through a 70-µm filter. After RBC lysis (RBC Lysis Buffer, Thermo Fisher Scientific) for 3 min, myelin was removed from the sample by Percoll (Sigma-Aldrich) centrifugation. Cells were then resuspended at 1 × 10^6^ ml^−1^ in 100 µl PBS and transferred to a 96-well plate. Before further staining, samples were resuspended in Zombie Aqua viability dye (1:400, BioLegend) and incubated for 30 min on ice.

For extracellular staining of collected or in vitro cultured cells, samples were incubated with Fc blocking solution (1:100 anti-mouse CD16/32 antibody, BioLegend, 101302) in FACS buffer (1× PBS with 2% FBS). After blocking, samples were incubated with antibodies (Supplementary Table 1) for 30 min on ice. Stained samples were then fixed with 2% formaldehyde in PBS on ice for 15 min.

Before acquisition, 10 µl of AccuCheck Counting Beads (Thermo Fisher Scientific) was added to each sample. To calculate the number of cells per gram of tumor, the following calculation was used: number of acquired cells × (number of input beads ÷ number of acquired beads) × (1 ÷ fraction of sample stained) × (1 ÷ tumor weight). Samples were acquired on an LSRFortessa (BD Biosciences) using FACSDiva software version 9 (BD Biosciences) and analyzed using FlowJo version 10 (BD Biosciences).

Mouse or human-specific antibodies were purchased from BD Biosciences, Invitrogen, MBL International, R&D Systems or BioLegend and are listed in Supplementary Table 1. Tissue processing and flow cytometry was performed as described previously^57^.

For CD107a staining, 1 × 10^5^ OT-1 T cells were co-cultured with 5 × 10^4^ OVA-loaded BMDMs, 1 × 10^4^ CT2A-OVA tumor cells or 1 × 10^4^ CT2A-TRP2-β2mKO tumor cells or cultured alone in TCM supplemented with IL-2 in 96-well plates. Macrophages were stained with CellTrace Violet. At the start of co-culture, anti-mouse CD107a antibody (Supplementary Table 1) was added, along with GolgiStop (2 µM; BD Biosciences) to prevent antibody breakdown. After 5 h of co-culture at 37 °C, cells were Fc blocked and then stained for CD8 (BV650, Supplementary Table 1), washed and fixed with formalin^58^.

### In vitro cytotoxicity assays

In vitro cytotoxicity assays were performed by co-culturing sorted CD8^+^ T cells (Miltenyi Biotec, 130-104-075) with tumor cells in the presence or absence of antigen-pulsed macrophages. Before co-culture, target cells were labeled with CellTrace Violet (Invitrogen) or CellTrace Far Red, and macrophages were labeled with CellTrace CFSE according to the manufacturer’s protocol to distinguish each cell type. TRP2 T cells, OT-1 T cells, DMF5 T cells or sham-transduced T cells were co-cultured with the designated target cell in T cell medium for 24 h in 96-well flat-bottom plates. For cytotoxicity experiments with murine T cells, 1 × 10^4^ total tumor cells, 1 × 10^5^ T cells and 5 × 10^4^ macrophages were added to each well. For cytotoxicity experiments with human T cells, 1 × 10^4^ total tumor cells and 1 × 10^4^ T cells were added to each well. For cytotoxicity experiments with mixed tumor cells, 5 × 10^3^ tumor cells of each line were added, for a total of 1 × 10^4^ tumor cells per well. After 24 h, cells were detached using trypsin and resuspended in 100 µl FACS buffer with 10 µl CountBright beads (Life Technologies Absolute Counting Beads, C36950) per well. Remaining viable tumor cells were quantified with flow cytometry. Percent lysis was calculated by counting remaining viable tumor cells in experimental wells versus tumor-only control wells, normalized as cells per bead and expressed as percent survival compared with tumor-only control wells (percent survival = ((experimental well viable cells ÷ bead count) ÷ (tumor-only well viable cells ÷ bead count)) × 100. In vitro cytotoxicity assays were performed with the following tumor cell lines: CT2A-β2mKO, CT2A-TRP2-β2mKO, CT2A-TRP2, GL261-OVA, GL261-OVA-β2mKO, YUMMER-FasKO, M202 and M202-β2mKO.

For cytotoxicity assays with blocking antibodies, 10 µg ml^−1^ anti-mouse NKG2D (clone HMG2D, BioXCell, BE0111), anti-mouse TRAIL (clone N2B2, Invitrogen, 16-5951-85), anti-mouse ICAM-1 (clone YN1/1.7.4, BioLegend, 116101), anti-mouse αLFA-1 (clone M17/4, BioLegend, 101118) or anti-human NKG2D (clone 1D11, BioXCell, BE0351) antibodies were added to T cells 30 min before co-culture with tumor cells. Remaining viable tumor cells were quantified after 24 h as described above.

For in vitro cytotoxicity involving TCR activation through the anti-CD3 antibody, mouse anti-CD3 (145-2C11, BioLegend, 100340) or human anti-CD3 (OKT3, BioLegend, 317326) antibodies were diluted in PBS to 1 µg ml^−1^ and then added to 96-well plates (100 µl per well). Plates were incubated at 5% CO_2_ and 37 °C for 2 h, and then anti-CD3 antibody was aspirated from the wells before the addition of cells.

### In vitro Transwell cytotoxicity assays

Transwell in vitro cytotoxicity experiments were performed as described above using 6.5-mm 0.4-µm and 5.0-µm Transwell inserts (Corning Costar; 0.4 µm, 3470; 5.0 µm, 3421) with the following modifications. A total of 1 × 10^5^ CT2A-OVA-β2mKO tumor cells were counted, stained with CellTrace Violet and plated on the bottom of 24-well plates. BMDMs were loaded with TRP2 peptide as described above and then stained with CFSE. TRP2 macrophages (5 × 10^4^) and TRP2-specific T cells (1 × 10^6^) were then plated in the top compartment of the Transwell at a 10:1 ratio of T cells to tumor cells and a 5:1 ratio of macrophages to tumor cells. After 24 h of culturing, cells were dislodged and quantified by flow cytometry. The inability of macrophages to pass through the 5.0-µm Transwell barrier was confirmed by the absence of CFSE-positive cells.

### RNA expression analysis

OT-1 T cells were isolated from OT-1 mouse splenocytes, which were activated and cultured as described above. OT-1 T cells were then co-cultured either alone in T cell medium supplemented with IL-2 (50 IU ml^−1^ IL-2), with OVA-loaded macrophages or with both OVA-loaded macrophages and CT2A-TRP2-β2mKO tumor cells. Cells were cultured at a 5:1 T cell-to-tumor ratio and a 2:1 T cell-to-macrophage ratio. After 24 h of co-culture, CD8^+^ T cells were sorted by flow cytometry, and RNA was extracted (RNeasy Mini Kit, Qiagen). RNA was analyzed on an nCounter MAX Analysis System (NanoString) with the PanCancer Immune Profiling Panel (NanoString) according to manufacturer instructions. Expression data were normalized and quality controlled using nSolver (version 4.0.70). Differential expression and gene set analysis were performed using nSolver Advanced Analysis software (version 2.0.134). Differential expression analysis *P* values were corrected for multiple testing using the Benjamini–Yekutieli false discovery rate.

### Analysis of NKG2D ligand expression within *B2M*-mutated tumors

Pan-Cancer Atlas mRNA Illumina HiSeq data were downloaded from the PanCanAtlas (TCGA), and samples were analyzed for mutations within the *B2M* gene. Analysis was performed using R (version 4.2.2). RNA sequencing data from patients with *B2M* mutations (*n* = 132) were compared to RNA sequencing data from 200 patients that were randomly subset from the remaining 10,327 patients within the PanCanAtlas dataset. Differential gene expression was evaluated using DeSeq2 (version 1.38.3). Differential expression analysis *P* values were calculated using the Wald test and corrected for multiple testing using the Benjamini–Hochberg false discovery rate. log_2_ (fold change) and adjusted *P* values for each gene were plotted using ggplot2 (version 3.4.2). NKG2DLs were specifically highlighted to assess expression of ligands in *B2M*-mutant compared to *B2M*-wild-type tumors.

### Statistics and reproducibility

Differential expression analyses were performed as stated above. Graphs represent mean ± s.e.m. unless otherwise stated. Statistical analysis was conducted in GraphPad Prism version 9.5.0 (GraphPad Software), primarily using two-tailed, unpaired *t*-tests, two-way analysis of variance (ANOVA) or one-way ANOVA to compare means across groups. For all experiments, a *P* value of <0.05 was considered statistically significant. No statistical methods were used to predetermine sample size. Sample sizes were instead determined based on historical sample sizes capable of detecting biologically significant differences for certain assays. If no historical data were available, pilot experiments were performed to determine the relative variability of the assay. Data were assumed to be normal, but this was not always formally tested.

The statistical tests employed for each data presentation and number of samples (*n*) are designated in the respective figure legends. Individual data points are represented as dots in graphs. Analyses were adjusted for multiple comparisons as indicated in figure legends. Kaplan–Meier curves were generated for survival analyses, and the two-sided Gehan–Breslow–Wilcoxon test was used to compare curves. No data were excluded from analysis. Mice that underwent treatment were randomized, within their genotype, to treatment groups after tumor injection. In vitro experiments were not randomized. All survival studies were monitored with the help of veterinary staff from the Duke animal facility who were blinded to the studies and reported endpoints accordingly. Other data collection and analysis were not performed blind to the conditions of the experiments and outcome assessment.

### Reporting summary

Further information on research design is available in the Nature Portfolio Reporting Summary linked to this article.

### Supplementary information

Supplementary Information Supplementary Table 1.

Reporting Summary

### Source data

Source Data Fig. 1 Statistical source data for Fig. 1.

Source Data Fig. 2 Statistical source data for Fig. 2.

Source Data Fig. 3 Statistical source data for Fig. 3.

Source Data Fig. 4 Statistical source data for Fig. 4.

Source Data Fig. 5 Statistical source data for Fig. 5.

Source Data Extended Data Fig. 1 Statistical source data for Extended Data Fig. 1.

Source Data Extended Data Fig. 3 Statistical source data for Extended Data Fig. 3.

Source Data Extended Data Fig. 4 Statistical source data for Extended Data Fig. 4.

Source Data Extended Data Fig. 5 Statistical source data for Extended Data Fig. 5.

## Data Availability

Gene expression data that support the findings of this study have been deposited in the Gene Expression Omnibus under accession code GSE220960. Human Pan-Cancer cohort RNA expression data were derived from the TCGA Research Network: https://portal.gdc.cancer.gov/about-data/publications/pancanatlas. The remaining data are available within the article or as source data. All other data supporting the findings of this study are available from the corresponding author upon reasonable request. Source data are provided with this paper.

## Code availability

No custom algorithms were used in this study. The R code that was used to perform differential expression analysis on the TCGA PanCan cohort can be found on Zenodo: 10.5281/zenodo.8007006.
