# Supplementary material for: CD8+ T cells maintain killing of MHC-I-negative tumor cells through the NKG2D–NKG2DL axis
Source: Nat Cancer. 2023 Aug 3;4(9):1258–72. doi: 10.1038/s43018-023-00600-4 (PMC10518253; doi:10.1038/s43018-023-00600-4)
Supplement: Supplementary file 1 — Supplementary Table 1. [file 43018_2023_600_MOESM1_ESM.pdf]

nature cancer

Article

https://doi.org/10.1038/s43018-023-00600-4

CD8$^+$ T cells maintain killing of MHC-I-negative tumor cells through the NKG2D–NKG2DL axis

In the format provided by the authors and unedited

Target	Clone	Source, Catalogue #	Fluorophore	Dilution
Murine CD8α	QA17A07	Biolegend 155005	APC	1:100
Murine CD8α	53-6.7	Biolegend 100705	FITC	1:100
Murine CD8α	53-6.7	Biolegend 100741	BV650	1:100
Murine CD107a	1D4B	Biolegend 121605	FITC	1:100
Murine CD45	30-F11	Biolegend 103139	BV605	1:100
Murine NKG2D	Cx5	Biolegend 130214	PE/Dazzle 594	1:100
Murine CD3	17A2	Biolegend 100227	BV421	1:100
Murine CD3	17A2	Biolegend 100235	APC	1:100
Murine CD4	RM4-5	Biolegend 100512	PE	1:100
Murine Fas-L	MFL3	Invitrogen 25-5911-82	PE/Cyanine7	1:100
Murine CD4	GK1.5	Biolegend 100434	PerCP/Cyanine5.5	1:100
Murine NKp46	29A1.4	Biolegend 137627	AF647	1:100
Murine RAE-1d	186107	BD Biosciences 748075	BB700	1:100
Murine ULBP-1/MULT-1	237104	R&D Systems FAB2588A	APC	1:100
Murine H2-Kb/H2-Kd	28-8-6	Biolegend 114607	PE	1:100
Human MICA/MICB	6D4	Biolegend 320907	APC	1:100
Human ULBP1	170818	R&D Systems FAB1380P	PE	1:100
Human ULBP3	166510	R&D Systems FAB1517P	PE	1:100
Murine Trp2 Tetramer<br>H-2Kb TRP-2 Tetramer-SVYDFFVWL		MBL International TB-5004-1	PE	1:100
Murine NK1.1	PK136	Biolegend 108718	AF488	1:100
Murine CD16/32 (Fc Block)	93	Biolegend 101302	Unconjugated	1:100
Zombie Aqua (Live/dead)	N/A	Biolegend 432102	BV510	1:400

CellTrace^{TM} Violet		Thermo Fisher C34557		Diluted per manufacturer's instructions
CellTrace^{TM} CFSE		Thermo Fisher C34554		Diluted per manufacturer's instructions
CellTrace^{TM} Far Red		Thermo Fisher C34564		Diluted per manufacturer's instructions
