# Supplementary material for: CD8+ T cells maintain killing of MHC-I-negative tumor cells through the NKG2D–NKG2DL axis
Source: Nat Cancer. 2023 Aug 3;4(9):1258–72. doi: 10.1038/s43018-023-00600-4 (PMC10518253; doi:10.1038/s43018-023-00600-4)
Supplement: Supplementary file 2 — Reporting Summary [file 43018_2023_600_MOESM2_ESM.pdf]

nature portfolio

Corresponding author(s): Peter Fecci

Last updated by author(s): 06/07/2023

Reporting Summary

Nature Portfolio wishes to improve the reproducibility of the work that we publish. This form provides structure for consistency and transparency in reporting. For further information on Nature Portfolio policies, see our Editorial Policies and the Editorial Policy Checklist.

Statistics

For all statistical analyses, confirm that the following items are present in the figure legend, table legend, main text, or Methods section.

n/a	Confirmed	
☐	☒	The exact sample size (*n*) for each experimental group/condition, given as a discrete number and unit of measurement
☐	☒	A statement on whether measurements were taken from distinct samples or whether the same sample was measured repeatedly
☐	☒	The statistical test(s) used AND whether they are one- or two-sided<br>*Only common tests should be described solely by name; describe more complex techniques in the Methods section.*
☒	☐	A description of all covariates tested
☐	☒	A description of any assumptions or corrections, such as tests of normality and adjustment for multiple comparisons
☐	☒	A full description of the statistical parameters including central tendency (e.g. means) or other basic estimates (e.g. regression coefficient) AND variation (e.g. standard deviation) or associated estimates of uncertainty (e.g. confidence intervals)
☐	☒	For null hypothesis testing, the test statistic (e.g. *F*, *t*, *r*) with confidence intervals, effect sizes, degrees of freedom and *P* value noted<br>*Give P values as exact values whenever suitable.*
☒	☐	For Bayesian analysis, information on the choice of priors and Markov chain Monte Carlo settings
☒	☐	For hierarchical and complex designs, identification of the appropriate level for tests and full reporting of outcomes
☒	☐	Estimates of effect sizes (e.g. Cohen's *d*, Pearson's *r*), indicating how they were calculated

Our web collection on statistics for biologists contains articles on many of the points above.

Software and code

Policy information about availability of computer code

Data collection	Flow cytometry data including cytotoxicity assays were collected using BD FACSDiva Software v.9. mRNA counts were measured using the NanoString nCounter platform.
Data analysis	Flow cytometry data were analyzed with Flowjo Software v.10 (BD).<br><br>Statistical analyses for in vitro and in vivo studies were performed with GraphPad Prism Software v9.5.0.<br><br>nSolver (v. 4.0.70) and nSolver Analysis Software (v. 2.0.134) (NanoString Technologies) were used for the analysis and quality control of mRNA data.<br><br>Differential expression analysis of TCGA PanCan Cohort was performed with R (v. 4.2.2) using DeSeq2 (v. 1.38.3), and the plot was generated using ggplot2 (v. 3.4.2).<br><br>The R code that was used to perform the differential expression analysis on the TCGA PanCan cohort can be found on Zenodo: https://doi.org/10.5281/zenodo.8007006

For manuscripts utilizing custom algorithms or software that are central to the research but not yet described in published literature, software must be made available to editors and reviewers. We strongly encourage code deposition in a community repository (e.g. GitHub). See the Nature Portfolio guidelines for submitting code & software for further information.

Data

Policy information about availability of data

All manuscripts must include a data availability statement. This statement should provide the following information, where applicable:

- Accession codes, unique identifiers, or web links for publicly available datasets
- A description of any restrictions on data availability
- For clinical datasets or third party data, please ensure that the statement adheres to our policy

Gene expression data that support the findings of this study have been deposited in the Gene Expression Omnibus (GEO) under accession code GSE220960.

The human Pan-Cancer cohort RNA expression data were derived from the TCGA Research Network: https://portal.gdc.cancer.gov/about-data/publications/pancanatlas.

The remaining data are available within the article or source data file. Source data are provided with this paper. All other data supporting the findings of this study are available from the corresponding author upon reasonable request.

Human research participants

Policy information about studies involving human research participants and Sex and Gender in Research.

Reporting on sex and gender	Not applicable
Population characteristics	Not applicable
Recruitment	Not applicable
Ethics oversight	Not applicable

Note that full information on the approval of the study protocol must also be provided in the manuscript.

Field-specific reporting

Please select the one below that is the best fit for your research. If you are not sure, read the appropriate sections before making your selection.

☒ Life sciences ☐ Behavioural & social sciences ☐ Ecological, evolutionary & environmental sciences

For a reference copy of the document with all sections, see nature.com/documents/nr-reporting-summary-flat.pdf

Life sciences study design

All studies must disclose on these points even when the disclosure is negative.

Sample size	Power analysis was not used to predetermine sample size. Sample sizes were instead determined based on historical sample sizes that were capable of detecting biologically significant differences for certain assays. If no historical data was available, pilot experiments were performed to determine the relative variability of the assay. All in vivo survival experiments were performed with at least 4 biologic replicates to ensure reproducibility. Sample sizes for in vivo experiments were chosen based on historical experience and were variable based on numbers of surviving mice available at experimental time-points or technical limitations.
Data exclusions	No data were excluded from analysis.
Replication	All major experiments were run with at least 3 biologic replicates, and attempts at experimental replication were successful.
Randomization	Mice were randomly assigned to treatment groups within a given genotype. Mice were randomized after intracranial injection and prior to treatment. In vitro groups were randomly assigned.
Blinding	For survival experiments, humane endpoint checks for mice were performed by an animal technician blinded to expected outcomes. For all other experiments, blinding was not possible due to personnel shortages not allowing for availability of separate investigators for data acquisition and analysis.

Reporting for specific materials, systems and methods

We require information from authors about some types of materials, experimental systems and methods used in many studies. Here, indicate whether each material, system or method listed is relevant to your study. If you are not sure if a list item applies to your research, read the appropriate section before selecting a response.

Materials & experimental systems

n/a	Involved in the study
☐	☒ Antibodies
☐	☒ Eukaryotic cell lines
☒	☐ Palaeontology and archaeology
☐	☒ Animals and other organisms
☒	☐ Clinical data
☒	☐ Dual use research of concern

Methods

n/a	Involved in the study
☒	☐ ChIP-seq
☐	☒ Flow cytometry
☒	☐ MRI-based neuroimaging

Antibodies

Antibodies used

Antibodies and dilutions used for flow cytometry are noted below:

Murine CD8α Clone QA17A07 Biolegend 155005 APC 1:100
Murine CD8α Clone 53-6.7 Biolegend 100705 FITC 1:100
Murine CD8α Clone 53-6.7 Biolegend 100741 BV650 1:100
Murine CD107a Clone 1D4B Biolegend 121605 FITC 1:100
Murine CD45 Clone 30-F11 Biolegend 103139 BV605 1:100
Murine NKG2D Clone Cx5 Biolegend 130214 PE/Dazzle 594 1:100
Murine CD3 Clone 17A2 Biolegend 100227 BV421 1:100
Murine CD3 Clone 17A2 Biolegend 100235 APC 1:100
Murine CD4 Clone RM4-5 Biolegend 100512 PE 1:100
Murine Fas-L Clone MFL3 Invitrogen 25-5911-82 PE/Cyanine7 1:100
Murine CD4 Clone GK1.5 Biolegend 100434 PerCP/Cyanine5.5 1:100
Murine NKp46 Clone 29A1.4 Biolegend 137627 AF647 1:100
Murine RAE-1d Clone 186107 BD Biosciences 748075 BB700 1:100
Murine ULBP-1/MULT-1 Clone 237104 R&D Systems FAB2588A APC 1:100
Murine H2-Kb/H2-Kd Clone 28-8-6 Biolegend 114607 PE 1:100
Human MICA/MICB Clone 6D4 Biolegend 320907 APC 1:100
Human ULBP1 Clone 170818 R&D Systems FAB1380P PE 1:100
Human ULBP3 Clone 166510 R&D Systems FAB1517P PE 1:100
Murine Trp2 Tetramer H-2Kb TRP-2 Tetramer-SVYDFFVWL, MBL International TB-5004-1 bPE 1:100
Murine NK1.1 Clone PK136 Biolegend 108718 AF488 1:100
Murine CD16/32 (Fc Block)Clone 93 Biolegend 101302 Unconjugated 1:100
Zombie Aqua (Live/dead) BioLegend 42310 BV 510 1:400

For functional blocking experiments: anti-mouse NKG2D (Clone HMG2D, BioXCell Catalog # BE0111), anti-mouse TRAIL (Clone N2B2, Invitrogen Catalog #16-5951-85) , anti-mouse ICAM-1 (Clone YN1/1.7.4, BioLegend Catalogue #116101), anti-mouse LFA-1 (Clone M17/4, Biolegend Catalogue # 101118), or anti-human NKG2D (Clone 1D11, BioXCell Catalogue #BE0351). All blocking antibodies and corresponding vendor-matched isotype controls were used at 10 ug/mL.

Detailed information from the manufacturers:
FFlow cytometry:
https://www.biolegend.com/en-us/products/apc-anti-mouse-cd8a-recombinant-antibody-16376?GroupID=BLG15858
https://www.biolegend.com/en-gb/products/fitc-anti-mouse-cd8a-antibody-153
https://www.biolegend.com/en-us/search-results/brilliant-violet-650-anti-mouse-cd8a-antibody-7635?GroupID=BLG2559
https://www.biolegend.com/de-at/products/fitc-anti-mouse-cd107a-lamp-1-antibody-3587
https://www.biolegend.com/en-us/products/brilliant-violet-605-anti-mouse-cd45-antibody-8721?GroupID=BLG6831
https://www.biolegend.com/en-us/products/pedazzle-594-anti-mouse-cd314-antibody-15542
https://www.biolegend.com/en-us/search-results/brilliant-violet-421-anti-mouse-cd3-antibody-7326
https://www.biolegend.com/en-us/products/apc-anti-mouse-cd3-antibody-8055?GroupID=BLG242
https://www.biolegend.com/nl-be/products/pe-anti-mouse-cd4-antibody-482
https://www.thermofisher.com/antibody/product/CD178-Fas-Ligand-Antibody-clone-MFL3-Monoclonal/25-5911-82
https://www.biolegend.com/en-gb/products/percp-cyanine5-5-anti-mouse-cd4-antibody-4220?GroupID=BLG4745
https://www.biolegend.com/en-us/search-results/alexa-fluor-647-anti-mouse-cd335-nkp46-antibody-12304?GroupID=BLG8849
https://www.bdbiosciences.com/en-us/products/reagents/flow-cytometry-reagents/research-reagents/single-color-antibodies-ruo/bb700-rat-anti-mouse-rae-1.748075
https://www.rndsystems.com/products/mouse-ulbp-1-mult-1-apc-conjugated-antibody-237104_fab2588a
https://www.biolegend.com/en-us/products/pe-anti-mouse-h-2k-b-h-2d-b-antibody-1686?GroupID=BLG2368
https://www.mblintl.com/products/tb-5004-1/
https://www.biolegend.com/en-us/search-results/alexa-fluor-488-anti-mouse-nk-1-1-antibody-3143?GroupID=GROUP20
https://www.biolegend.com/en-us/products/purified-anti-mouse-cd16-32-antibody-190?GroupID=BLG9237
https://www.biolegend.com/en-ie/products/apc-anti-human-mica-micb-antibody-3065
https://www.rndsystems.com/products/human-ulbp-1-pe-conjugated-antibody-170818_fab1380p?gclid=EAIaIQobChMI5YWOkeGs_wIVEEFyCh14vQcQEAAYASAAEgKtffD_BwE&gclsrc=aw.ds
https://www.rndsystems.com/products/human-ulbp-3-pe-conjugated-antibody-166510_fab1517p?

gclid=EAIaIQobChMI_dSM_OGs_wIVVENyCh0bIAG7EAAYASAAEgKh8fD_BwE&gclsrc=aw.ds
https://www.thermofisher.com/order/catalog/product/C34557
https://www.thermofisher.com/order/catalog/product/C34554
https://www.thermofisher.com/order/catalog/product/C34564
https://www.biolegend.com/en-us/products/zombie-aqua-fixable-viability-kit-8444?GroupID=BLG2181

Functional blocking:
https://bioxcell.com/invivomab-anti-mouse-nkg2d-be0111
https://www.thermofisher.com/antibody/product/CD253-TRAIL-Antibody-clone-N2B2-Monoclonal/16-5951-85
https://www.biolegend.com/fr-lu/products/ultra-leaf-purified-anti-mouse-cd54-antibody-18350
https://www.biolegend.com/en-us/products/ultra-leaf-purified-anti-mouse-cd11a-antibody-8079
https://bioxcell.com/invivomab-anti-human-nkg2d-cd314-be0351

Validation

Each primary antibody has been validated by the manufacturer for use to detect the indicated murine or human targets. Blocking antibodies were reported by the manufacturer as functionally blocking. All antibodies are from commercial sources and have been validated by the vendors and validation materials are available on the appropriate websites from vendors (Biolegend, Thermo Fisher, MBL International, Invitrogen, R&D Systems). All antibodies utilized in this manuscript were optimized by titrating to achieve a maximal staining index.

Eukaryotic cell lines

Policy information about cell lines and Sex and Gender in Research

Cell line source(s)

Murine cell lines studied included CT2A malignant glioma, GL261 malignant glioma, B16-F10 melanoma (B16), and YUMMER melanoma. The CT2A line was provided by Robert L. Martuza (Massachusetts General Hospital) and the GL261 line was provided by the National Cancer Institute. These cell lines are additionally available commercially (CT2A, Sigma-Aldrich, SCC194; GL261, DSMZ, ACC 802). The B16-F10 cell line was a gift from J. Sampson. The YUMMER-FasKO cell line was a gift from K. Wood. All cell lines are syngeneic in C57BL/6 mice. Human cell lines used were M202 and M202 B2mKO, both a gift from A. Ribas, and HEK 293T cells, a gift from J. Sampson. Murine adult primary dermal fibroblasts (C57BL/6) cells were obtained from Cell Biologics (Catalog #C57-6067).

Authentication

All cell lines were authenticated and tested negative for mycoplasma, and interspecies contamination by IDEXX Laboratories. (Westbrook, ME). Cell lines were authenticated by using NIST published 9 species-specific STR markers to establish genetic profiles. Interspecies contamination check for human, mouse, rat, African green monkey and Chinese hamster was also performed for each cell line.

Mycoplasma contamination

All cell lines were confirmed to be mycoplasma negative by IDEXX Laboratories.

Commonly misidentified lines
(See ICLAC register)

No commonly misidentified lines were used in this study.

Animals and other research organisms

Policy information about studies involving animals; ARRIVE guidelines recommended for reporting animal research, and Sex and Gender in Research

Laboratory animals

The Institutional Animal Care and Use Committee (IACUC) at Duke University approved all experimental procedures. Animal experiments Experiments were conducted on age and sex matched female mice between 8-12 weeks of age. C57BL/6 mice were purchased from Charles River Laboratories. OT-1 (#003831), CCR2KO (#004999), and CD8KO (#002665) were purchased from Jackson Laboratories. Animals were maintained under pathogen-free conditions, in temperature and humidity controlled housing, with free access to food and water, under a 12-h light/dark cycle at the Cancer Center Isolation Facility of Duke University Medical Center.

Wild animals

This study did not use wild animals.

Reporting on sex

Female mice were used for in vivo survival experiments to avoid unwanted immunogenicity related to sex chromosomes as syngeneic tumor lines were derived from female animals. Sex was not considered in the study design.

Field-collected samples

This study did not involve samples collected from the field.

Ethics oversight

All mouse experiments were approved by the Institutional Animal Care and Use Committee at Duke University Medical Center (protocol A163-21-08). The maximum subcutaneous tumor size permitted by Duke University Medical Center IACUC is 2000 mm3. This maximum tumor size was never exceeded in the studies.

Note that full information on the approval of the study protocol must also be provided in the manuscript.

Flow Cytometry

Plots

Confirm that:

☒ The axis labels state the marker and fluorochrome used (e.g. CD4-FITC).

☒ The axis scales are clearly visible. Include numbers along axes only for bottom left plot of group (a 'group' is an analysis of identical markers).

☒ All plots are contour plots with outliers or pseudocolor plots.

☒ A numerical value for number of cells or percentage (with statistics) is provided.

Methodology

Sample preparation	Sample preparation is extensively detailed here (PMID: 34597618). In brief, blood, spleen, and tumor were collected at day 17 post tumor implantation. Briefly, tissues were processed in RPMI, minced into single cell suspensions, cell-strained, counted, stained with antibodies, and analyzed via flow cytometry. Blood samples were directly labeled with antibodies and red blood cells subsequently lysed using eBioscience RBC lysis buffer (eBioscience, San Diego, CA) or BD Pharm Lyse (BD Biosciences). Spleen and tumor samples were subjected to RBC lysis prior to antibody-labeling.<br><br>For CD107a staining, 1x105 OT-1 T cells were co-cultured with 5x104 OVA loaded BMDMs, 1x104 CT2A-OVA tumor, 1x104 CT2A-TRP2-B2mKO tumor, or alone in TCM supplemented with IL-2 in 96 well plates. Macrophages were stained with CellTraceTM Violet. At the start of co-culture, anti-mouse CD107a antibody (Supplementary Table 1) was added, along with Golgistop (2µM; BD Biosciences) to prevent antibody breakdown. After 5h of co-culture at 37 degrees Celsius, the cells were Fc blocked then stained for CD8 (BV650, table 1), washed, and formalin fixed
Instrument	Analysis was performed with a BDLSR Fortessa Cell analyzer (BD Biosciences). Sorting was performed with a Sony SH800.
Software	Flow cytometry data was collected with FACS Diva Software v.9 (BD Biosciences) and analyzed using FlowJo v.10 (BD Biosciences). Sorting performed with Sony SH800 Software.
Cell population abundance	Cell isolation of CD8+ T cells was accomplished using negative selection beads (Miltenyi Biotec Cat. 130-104-075) or via FACS sorting with a Sony SH800. The purity of sorted cells was confirmed by flow cytometry and verified to be over 90%.
Gating strategy	The primary gating strategy is provided in Extended Data 4k. Live cells were gated using live/dead staining. FSC-A/FSC-H plots were used to determine singlet gates. FSC-A/SSC-A plots were used to determine cell population gates. In vitro panels included CellTrace stains for gating in/ out cell populations of interest when applicable. Positive and negative gates were determined based on isotype, untreated, or controls. In vivo tumor infiltrating lymphocyte panels were gated using fluorescence minus one (FMO) controls.

☒ Tick this box to confirm that a figure exemplifying the gating strategy is provided in the Supplementary Information.
